# Supplementary material for: Immunomodulatory drug fingolimod (FTY720) restricts the growth of opportunistic yeast Candida albicans in vitro and in a mouse candidiasis model
Source: PLoS One. 2022 Dec 7;17(12):e0278488. doi: 10.1371/journal.pone.0278488 (PMC9728862; doi:10.1371/journal.pone.0278488)
Supplement: S1 File — (DOCX) [file pone.0278488.s001.docx]

Immunomodulatory drug fingolimod (FTY720) restricts the growth of opportunistic yeast *Candida albicans in vitro* and in a mouse candidiasis model*:* Supplemental content

eTable 1; Summary of all of the comparisons in the study. Abbreviations: IFNg, interferon-gamma; diff., difference; C. alb, Candida albicans; IL10, interleukin-10; WBC, white blood cell; ALC, absolute lymphocyte count; CC, colony count.

| Outcome | Comparison | Effect measure | Value (standard error) | P value |
| --- | --- | --- | --- | --- |
| IFNγ | Control (Arm 1) vs. FTY720 (Arm 2) | Log_10_ Mean diff. | 0.13 (0.029) | 0.0004 |
|  | Control (Arm 1) vs. C. albicans (Arm 3) | Log_10_ Mean diff. | -0.11 (0.029) | 0.0016 |
|  | FTY720 (Arm 2) vs. C. alb before FTY720 (arm 4) | Log_10_ Mean rank diff. | -26 (NA) | <0.0001 |
|  | FTY720 (Arm 2) vs. C. alb with FTY720 (arm 5) | Log_10_ Mean rank diff. | -19 (NA) | 0.0026 |
|  | FTY720 (Arm 2) vs. C. alb after FTY720 (arm 6) | Log_10_ Mean rank diff. | -6.9 (NA) | 0.2784 |
|  | C. albicans (Arm 3) vs. C. alb before FTY720 (arm 4) | Log_10_ Mean rank diff. | -3.2 (NA) | 0.6135 |
|  | C. albicans (Arm 3) vs. C. alb with FTY720 (arm 5) | Log_10_ Mean rank diff. | 3.9 (NA) | 0.5434 |
|  | C. albicans (Arm 3) vs. C. alb after FTY720 (arm 6) | Log_10_ Mean rank diff. | 16 (NA) | 0.0113 |
| IL10 | Control (Arm 1) vs. FTY720 (Arm 2) | Log_10_ Mean diff. | -0.11 (0.035) | 0.0080 |
|  | Control (Arm 1) vs. C. albicans (Arm 3) | Log_10_ Mean diff. | 0.053 (0.035) | 0.1482 |
|  | FTY720 (Arm 2) vs. C. alb before FTY720 (arm 4) | Log_10_ Mean diff. | 0.16 (0.043) | 0.0008 |
|  | FTY720 (Arm 2) vs. C. alb with FTY720 (arm 5) | Log_10_ Mean diff. | 0.035 (0.043) | 0.4194 |
|  | FTY720 (Arm 2) vs. C. alb after FTY720 (arm 6) | Log_10_ Mean diff. | -0.042 (0.043) | 0.3289 |
|  | C. albicans (Arm 3) vs. C. alb before FTY720 (arm 4) | Log_10_ Mean diff. | -0.0055 (0.043) | 0.8970 |
|  | C. albicans (Arm 3) vs. C. alb with FTY720 (arm 5) | Log_10_ Mean diff. | -0.13 (0.043) | 0.0052 |
|  | C. albicans (Arm 3) vs. C. alb after FTY720 (arm 6) | Log_10_ Mean diff. | -0.20 (0.043) | <0.0001 |
| WBC | Control (Arm 1) vs. FTY720 (Arm 2) | Log_10_ Mean diff. | 0.24 (0.052) | 0.0004 |
|  | Control (Arm 1) vs. C. albicans (Arm 3) | Log_10_ Mean diff. | -0.18 (0.052) | 0.0038 |
|  | FTY720 (Arm 2) vs. C. alb before FTY720 (arm 4) | Log_10_ Mean diff. | -0.0033 (0.075) | 0.9653 |
|  | FTY720 (Arm 2) vs. C. alb with FTY720 (arm 5) | Log_10_ Mean diff. | -0.52 (0.075) | <0.0001 |
|  | FTY720 (Arm 2) vs. C. alb after FTY720 (arm 6) | Log_10_ Mean diff. | -0.10 (0.075) | 0.1920 |
|  | C. albicans (Arm 3) vs. C. alb before FTY720 (arm 4) | Log_10_ Mean diff. | 0.41 (0.075) | <0.0001 |
|  | C. albicans (Arm 3) vs. C. alb with FTY720 (arm 5) | Log_10_ Mean diff. | -0.11 (0.075) | 0.1641 |
|  | C. albicans (Arm 3) vs. C. alb after FTY720 (arm 6) | Log_10_ Mean diff. | 0.32 (0.075) | 0.0002 |
| ALC | Control (Arm 1) vs. FTY720 (Arm 2) | Log_10_ Mean diff. | 0.49 (0.047) | <0.0001 |
|  | Control (Arm 1) vs. C. albicans (Arm 3) | Log_10_ Mean diff. | -0.17 (0.048) | 0.0138 |
|  | FTY720 (Arm 2) vs. C. alb before FTY720 (arm 4) | Log_10_ Mean diff. | 0.10 (0.082) | 0.2253 |
|  | FTY720 (Arm 2) vs. C. alb with FTY720 (arm 5) | Log_10_ Mean diff. | -0.73 (0.082) | <0.0001 |
|  | FTY720 (Arm 2) vs. C. alb after FTY720 (arm 6) | Log_10_ Mean diff. | 0.13 (0.082) | 0.1097 |
|  | C. albicans (Arm 3) vs. C. alb before FTY720 (arm 4) | Log_10_ Mean diff. | 0.76 (0.082) | <0.0001 |
|  | C. albicans (Arm 3) vs. C. alb with FTY720 (arm 5) | Log_10_ Mean diff. | -0.077 (0.082) | 0.3536 |
|  | C. albicans (Arm 3) vs. C. alb after FTY720 (arm 6) | Log_10_ Mean diff. | 0.79 (0.082) | <0.0001 |
| CC –Kidney | C. albicans (arm 3) vs. C. alb before FTY720 (arm 4) | Mean diff. | 165 (38) | 0.0010 |
|  | C. albicans (arm 3) vs. C. alb with FTY720 (arm 5) | Mean diff. | 329 (17) | <0.0001 |
|  | C. albicans (arm 3) vs. C. alb after FTY720 (arm 6) | Mean diff. | 109 (32) | 0.0044 |
| CC –Liver | C. albicans (arm 3) vs. C. alb before FTY720 (arm 4) | Mean diff. | 112 (57) | 0.0690 |
|  | C. albicans (arm 3) vs. C. alb with FTY720 (arm 5) | Mean diff. | 297 (33) | 0.0002 |
|  | C. albicans (arm 3) vs. C. alb after FTY720 (arm 6) | Mean diff. | 127 (42) | 0.0114 |
| CC –Vagina | C. albicans (arm 3) vs. C. alb before FTY720 (arm 4) | Mean rank diff. | 8.1 (NA) | 0.1195 |
|  | C. albicans (arm 3) vs. C. alb with FTY720 (arm 5) | Mean rank diff. | 6.9 (NA) | 0.1849 |
|  | C. albicans (arm 3) vs. C. alb after FTY720 (arm 6) | Mean rank diff. | 14 (NA) | 0.0090 |
