# Supplementary material for: Immunomodulatory drug fingolimod (FTY720) restricts the growth of opportunistic yeast Candida albicans in vitro and in a mouse candidiasis model
Source: PLoS One. 2022 Dec 7;17(12):e0278488. doi: 10.1371/journal.pone.0278488 (PMC9728862; doi:10.1371/journal.pone.0278488)
Supplement: Attachment — Submitted filename: Comments.pdf [file pone.0278488.s002.pdf]

**Immunomodulatory drug fingolimod (FTY720) restricts the growth of opportunistic yeast Candida albicans in vitro and in a mouse candidiasis model**

Manuscript Id: PONE-D-22-15968

Comments after review:

1. The authors are advised to go through the entire manuscript and correct it for grammatical errors.
2. Authors should mention protocol number approved by Institutional Ethics Committee (IEC) in the manuscript.
3. In *in-vitro* well diffusion assay, how did the authors manage to load 200ml of sample in the wells. Also, please mention the zone of inhibition values obtained.
4. Please mention the histopathological images of livers, kidneys and vagina obtained after 21 days of treatment to provide visual analysis of Candida colonization between the treated and control groups.
5. Authors have mentioned that Fingolimod restricts the growth of opportunistic yeast Candida albicans in vitro and in a mouse candidiasis model. Have the authors investigated the *in vitro* effect of this compound on important virulence traits mainly- hydrolytic enzymes, yeast to hyphae transition, adhesion and biofilm formation. If yes, please mention this in manuscript.
