# Supplementary material for: Immunomodulatory drug fingolimod (FTY720) restricts the growth of opportunistic yeast Candida albicans in vitro and in a mouse candidiasis model
Source: PLoS One. 2022 Dec 7;17(12):e0278488. doi: 10.1371/journal.pone.0278488 (PMC9728862; doi:10.1371/journal.pone.0278488)
Supplement: Attachment — Submitted filename: oct-PONE-D-22-15968_R2_reviewer.pdf [file pone.0278488.s006.pdf]

PLOS ONE

Immunomodulatory drug fingolimod (FTY720) restricts the growth of opportunistic yeast Candida albicans in vitro and in a mouse candidiasis model

--Manuscript Draft--

**Manuscript Number:**	PONE-D-22-15968R2
**Article Type:**	Research Article
**Full Title:**	Immunomodulatory drug fingolimod (FTY720) restricts the growth of opportunistic yeast Candida albicans in vitro and in a mouse candidiasis model
**Short Title:**	Characterization of the antifungal effect of fingolimod (FTY720)
**Corresponding Author:**	Nahad Sedaghat<br>Isfahan University of Medical Sciences<br>Isfahan, IRAN, ISLAMIC REPUBLIC OF
**Keywords:**	Fingolimod; Candida albicans; candidiasis; Drug Repositioning.
**Abstract:**	Fingolimod (FTY720) is a drug derived from the fungicidal compound myriocin. As it was unclear whether FTY720 has antifungal effects as well, we aimed to characterize its effect on Candida (C.) albicans in vitro and in a mouse candidiasis model. In-vitro antifungal susceptibility testing was performed, along with a randomized, six-arm, parallel, open-label trial on 48 mice receiving oral FTY720 (0.3mg/kg/day), intraperitoneal C. albicans inoculum, or placebos with different combinations and chorological patterns. Immune response measures including interleukin-10, interferon-gamma, and absolute lymphocyte count, and fungal burden values including C. albicans colony counts in liver, kidney, and vagina were measured as outcomes. Microbroth dilution assay revealed FTY720's minimum inhibitory concentration (MIC 99) to be 0.25mg/mL for Candida albicans . Significant (P<0.05) reductions in fungal burden values of the infected mice receiving FTY720 were observed. As expected, the immune response measures were shifted towards an anti-inflammatory profile in the mice receiving FTY720. Since FTY720's MIC 99 is not reached in vivo with the regimen we used, we hypothesize that FTY720 synergizes the host's innate immune functions by inducing the production of reactive oxygen species. Further studies are warranted to unveil the mechanistic explanations of our observations, and clarify further aspects of repurposing FTY720 for antifungal use in clinical settings.
**Order of Authors:**	Niloofar Najarzadegan
	Mahboubeh Madani
	Masoud Etemadifar
	Nahad Sedaghat
**Response to Reviewers:**	Reviewer #1:<br>1.Scientific notation of writing Candida albicans is not correct in abstract. Please check. It should be uniform throughout the manuscript.<br>Response: Thank you for noticing. We uniformly changed all the instances in the abstract to "Candida albicans".<br>2.Please incorporate the images of well diffusion assay in the manuscript.<br>Response: Thank you for your suggestion. All eFigures were moved to the main manuscript.<br>3.Authors should keep space between values and their units throughout the manuscript and used SI units.<br>Response: Thank you for mentioning. We added the required space between the values and units. About using SI units, as far as we checked, we did not use non-SI units anywhere in the manuscript. In case we overlooked an instance of using any non-SI unit, we would appreciate if you could point to the line, so we could correct that instance.<br>4.Authors are advised to incorporate images to get better picture of their results.<br>Response: Thank you for your suggestion. As mentioned, we moved all the eFigures to the main manuscript. We unfortunately have a limitation in presenting further images apart from what is already presented, as the experiments are no longer in progress. We mentioned this point in the limitations section and advised future studies to account

Powered by Editorial Manager® and ProduXion Manager® from Aries Systems Corporation

for this limitation.
5.Please incorporate toxicity related studies in the manuscript. If already published by others cite that reference in the manuscript.
Response: Thank you for your attention. We added and discussed toxicity related studies as well in the revised manuscript. We hope the changes and improvements satisfy your concerns.

Reviewer #2:
0.The authors have answered have all the questions raised by me.
Response: We are pleased to hear that. Thank you for dedicating your time to our manuscript.
1.However, as the authors mentioned in the article that there are some significant limitations to the reproducibility of the experiments.
Response: Thank you for mentioning your concern about the replicability of the study and reproducibility of the results. In case your concern is due to the lack of standard photographic documentation of the experiments and results, we made the following efforts to account for this limitation and make our report of merit for publication. First of all, we described and disclosed all of the experiments in full detail and in complete accordance with the EQUATOR guidelines, in order to allow their complete repetition by any scientist across the globe. Therefore, replication of the experiments and reproduction of the results would be possible for any team concerned about the lack of proper photographic documentations in the present report. Additionally, all of the experiments were performed in compliance with strict regulations and were being monitored through surveillance systems by independent scientific, safety, and ethical review boards, consisting of anonymous nationally-certified scientists. Finally, we shared and deposited our data for all researchers across the globe to review and validate. We would also welcome any further suggestions to improve our work.
2. The supplementary figures are also not as per scientific standards.
Response: Thank you for mentioning your concern. We completely agree that the presented figures are not in compliance with scientific standards, as we unfortunately, did not intend to keep scientific photographic documentations at the beginning. The reason was that in presence of strict regulations and standard guidelines, we erroneously considered photographic documentations to be optional. Now, as the experiments are no longer in process, we are unfortunately limited in terms of presenting standard figures. However, describing the experiments with full detail allows their repetition by any researcher concerned about the reproducibility of the results. In the limitations section, we explicitly stated this point, and strongly encouraged further replication of the experiments using the detailed description of the methods we provided. We further stated that due to our limitation in providing standard figures, our conclusions are subject to reproduction of the results by future studies with standard photographic documentations.
3.especially handling microbiological Petri dishes without gloves is a serious concern.
Response: Thank you for your punctiliousness. We completely acknowledge your concern. We should mention that the photograph you are referring to was taken after completion of the experiments and documentation of their results. During the experiments, the laboratory researchers followed strict regulatory standards in terms of their safety; microbiological Petri dishes were not handled without gloves during any of the experiments, and the laboratory researchers wore sterile gowns, protective face-shields and masks, as well as following standard hand-washing protocols with povidone iodine solution before entering the laboratory. This has been evidenced by absence of biological and non-biological contamination in all of the Petri dishes except for one, and by the approval of the safety procedures by the regulatory scientific review board. These points were clarified in the revised manuscript and in the legend of the mentioned figure to appreciate any concern.
4.There are still a few typos in the manuscript (eg. Line 63 homo-, IFNg instead of γ in the etable etc.).
Response: We apologize for overseeing the mentioned typos in our reviews. The Latin phrase “in homo” means “in man” in English; we exchanged “in homo” with the standard English term “in humans” to avoid any confusion. IFNg was changed to IFNγ in the supplementary table as well. Furthermore, we reviewed the revised manuscript several more times to ensure proper usage of language for the readers. We would certainly appreciate any further suggestions for improving our report.
5.Therefore, I recommend to resubmit the article with proper figures.

	Response: Thank you for your recommendation. As mentioned previously, the experiments are no longer in progress, therefore, we are unfortunately limited in presenting standard photographic documentations. We truly hope our explicit and detailed disclosure of the experimental procedures according to the EQUATOR guidelines and encouragement of future replicative studies could at least, partially resolve your concerns about the reproducibility of the results. We are also, hoping that the publication of our study, if considered of merit based on novelty and relevance, leads to conduction of further studies on the subject accounting for our limitations (including provision of standard figures) and validating the results. Once again, we would like to appreciate your punctiliousness and explicitly in critically appraising our study, and thank you for your time and consideration.
**Additional Information:**	
**Question**	**Response**
**Financial Disclosure**<br><br>Enter a financial disclosure statement that describes the sources of funding for the work included in this submission. Review the submission guidelines for detailed requirements. View published research articles from *PLOS ONE* for specific examples.<br><br>This statement is required for submission and **will appear in the published article** if the submission is accepted. Please make sure it is accurate.<br><br>**Unfunded studies**<br>Enter: *The author(s) received no specific funding for this work.*<br><br>**Funded studies**<br>Enter a statement with the following details:<br>• Initials of the authors who received each award<br>• Grant numbers awarded to each author<br>• The full name of each funder<br>• URL of each funder website<br>• Did the sponsors or funders play any role in the study design, data collection and analysis, decision to publish, or preparation of the manuscript?<br>• **NO** - Include this sentence at the end of your statement: *The funders had no role in study design, data collection and analysis, decision to publish, or preparation of the manuscript.*<br>• **YES** - Specify the role(s) played.<br><br>* typeset	The author(s) received no specific funding for this work.
**Competing Interests**	The authors have declared that no competing interests exist.

Powered by Editorial Manager® and ProduXion Manager® from Aries Systems Corporation

Use the instructions below to enter a competing interest statement for this submission. On behalf of all authors, disclose any competing interests that could be perceived to bias this work—acknowledging all financial support and any other relevant financial or non-financial competing interests.<br><br>This statement is required for submission and **will appear in the published article** if the submission is accepted. Please make sure it is accurate and that any funding sources listed in your Funding Information later in the submission form are also declared in your Financial Disclosure statement.<br><br>View published research articles from *PLOS ONE* for specific examples.<br><br>**NO authors have competing interests**<br><br>Enter: *The authors have declared that no competing interests exist.*<br><br>**Authors with competing interests**<br><br>Enter competing interest details beginning with this statement:<br><br>*I have read the journal's policy and the authors of this manuscript have the following competing interests: [insert competing interests here]*<br><br>* typeset	
**Ethics Statement**<br><br>Enter an ethics statement for this submission. This statement is required if the study involved:<br><br>• Human participants<br>• Human specimens or tissue<br>• Vertebrate animals or cephalopods<br>• Vertebrate embryos or tissues<br>• Field research	This study was approved by the ethics committee of the Islamic Azad University, Falavarjan Branch.

Write "N/A" if the submission does not require an ethics statement.

General guidance is provided below. Consult the submission guidelines for detailed instructions. **Make sure that all information entered here is included in the Methods section of the manuscript.**

Format for specific study types

Human Subject Research (involving human participants and/or tissue)

- Give the name of the institutional review board or ethics committee that approved the study
- Include the approval number and/or a statement indicating approval of this research
- Indicate the form of consent obtained (written/oral) or the reason that consent was not obtained (e.g. the data were analyzed anonymously)

Animal Research (involving vertebrate animals, embryos or tissues)

- Provide the name of the Institutional Animal Care and Use Committee (IACUC) or other relevant ethics board that reviewed the study protocol, and indicate whether they approved this research or granted a formal waiver of ethical approval
- Include an approval number if one was obtained
- If the study involved *non-human primates*, add *additional details* about animal welfare and steps taken to ameliorate suffering
- If anesthesia, euthanasia, or any kind of animal sacrifice is part of the study, include briefly which substances and/or methods were applied

Field Research

Include the following details if this study involves the collection of plant, animal, or other materials from a natural setting:

- Field permit number
- Name of the institution or relevant body that granted permission

Powered by Editorial Manager® and ProduXion Manager® from Aries Systems Corporation

**Data Availability**<br><br>Authors are required to make all data underlying the findings described fully available, without restriction, and from the time of publication. PLOS allows rare exceptions to address legal and ethical concerns. See the PLOS Data Policy and FAQ for detailed information.<br><br>A Data Availability Statement describing where the data can be found is required at submission. Your answers to this question constitute the Data Availability Statement and **will be published in the article**, if accepted.<br><br>**Important:** Stating 'data available on request from the author' is not sufficient. If your data are only available upon request, select 'No' for the first question and explain your exceptional situation in the text box.<br><br>Do the authors confirm that all data underlying the findings described in their manuscript are fully available without restriction?	Yes - all data are fully available without restriction
**Describe where the data may be found in full sentences. If you are copying our sample text, replace any instances of XXX with the appropriate details.**<br><br>• If the data are **held or will be held in a public repository**, include URLs, accession numbers or DOIs. If this information will only be available after acceptance, indicate this by ticking the box below. For example: *All XXX files are available from the XXX database (accession number(s) XXX, XXX.*).<br>• If the data are all contained **within the manuscript and/or Supporting Information files**, enter the following: *All relevant data are within the manuscript and its Supporting Information files.*<br>• If neither of these applies but you are able to provide **details of access elsewhere**, with or without limitations, please do so. For example:	All relevant data are within the manuscript and its Supporting Information files.

Powered by Editorial Manager® and ProduXion Manager® from Aries Systems Corporation

Data cannot be shared publicly because of [XXX]. Data are available from the XXX Institutional Data Access / Ethics Committee (contact via XXX) for researchers who meet the criteria for access to confidential data.<br><br>*The data underlying the results presented in the study are available from (include the name of the third party and contact information or URL).*<br>• This text is appropriate if the data are owned by a third party and authors do not have permission to share the data.<br><br>* typeset	
Additional data availability information:	

Powered by Editorial Manager® and ProduXion Manager® from Aries Systems Corporation

Manuscript (revised, clean)

Click here to access/download;Manuscript;ms v1.3 Pone clean.docx

Immunomodulatory drug fingolimod (FTY720) restricts the growth of opportunistic yeast *Candida albicans in vitro* and in a mouse candidiasis model

Short Title: Characterization of the antifungal effect of fingolimod (FTY720)

Niloofar Najarzadegan[a]; Mahboubeh Madani[a]; Masoud Etemadifar[b]; Nahad Sedaghat[b,c]*.*

a. *Department of Microbiology, Falavarjan Branch, Islamic Azad University, Isfahan, Iran.*
b. *Alzahra Research Institute, Alzahra University Hospital, Isfahan University of Medical Sciences, Isfahan, Iran.*
c. *Network of Immunity in Infection, Malignancy, and Autoimmunity (NIIMA), Universal Scientific, Education, and Research Network (USERN), Isfahan, Iran.*

**Address for Correspondence:*

Mahboubeh Madani

Department of Microbiology, Falavarjan Branch, Islamic Azad University, Isfahan, Iran.

Email: mmadani66@gmail.com

Nahad Sedaghat

Alzahra Research Institute, Alzahra University Hospital, Isfahan University of Medical Sciences, Isfahan, Iran.

Email: nahad.sedaghat@gmail.com

Manuscript details:

- Word count (main text): 4273
- Number of figures: 7
- Number of tables: 0
- Number of supplemental files: 1
- Number of references: 42

Abstract

Fingolimod (FTY720) is a drug derived from the fungicidal compound myriocin. As it was unclear whether FTY720 has antifungal effects as well, we aimed to characterize its effect on *Candida albicans in vitro* and in a mouse candidiasis model. In vitro antifungal susceptibility testing was performed, along with a randomized, six-arm, par[illegible]al on 48 mice receiving oral FTY720 (0.3 mg/kg/day), intraperitoneal *Candida albicans* inoculum, or placebos with different combinations and chorological patterns. Immune response measures including interleukin-10, interferon-gamma, and absolute lymphocyte count, and fungal burden values including *Candida albicans* colony [illegible] in liver, kidney, and vagina were measured as outcomes. Microbroth dilution assay revealed FT[illegible]mum inhibitory concentration (MIC_{99}) to be 0.25 mg/mL for *Candida albicans*. Significant ($P<0.05$) reductions in fungal burden values of the infected mice receiving FTY720 were observed. As expected, the immune response measures were shifted towards an anti-inflammatory profile in the mice receiving FTY720. Since FTY720's MIC_{99} is not reached *in vivo* with the regimen we used, we hypothesize that FTY720 synergizes the host's innate immune functions by inducing the production of reactive oxygen species. Further studies are warranted to unveil the mechanistic explanations of our observations, and clarify further aspects of repurposing FTY720 for antifungal use in clinical settings.

Key words: Fingolimod; Candida albicans; candidiasis; Drug Repositioning.

1. Introduction

Fingolimod (FTY720) is an immunomodulatory drug currently indicated and approved by the FDA for treatment of the relapsing forms of multiple sclerosis (MS) – an autoimmune entity involving the CNS. It is used off-label for progressive MS and other autoimmune neuropathies, while being evaluated for extra-nervous pathologies as well (2-4). FTY720 is considered a prodrug of its phosphorylated form fingolimod-phosphate (FTY720-P). Being a modulator of the sphingosine-1-phosphate receptors (S1PR) by structural analogy, FTY720-P induces internalization of S1PR from the surface of lymphocytes, hindering their trafficking outside the primary lymphatics and, therefore, keeping them away from inflammatory sites in end organs (1).

While being associated with beneficial outcomes and reasonable tolerability among people with immune-mediated conditions such as MS (2, 3), safety and toxicity studies have shown both dose-dependent and time-dependent adverse effects associated with FTY720 (4). The time-dependent adverse effects have been opportunistic infections, reactivation of latent viruses, malignancies, etc., and were mostly deemed to be due to chronic immunosuppression (4). Among the dose-dependent adverse effects, the most common has been cardiotoxicity (2, 3). S1PRs are highly expressed on cardiomyocytes; the initial agonistic effect of FTY720-P on S1PRs before inducing their internalization seems to be responsible for its dose-dependent cardiotoxic effects (5). Hepatotoxic dose-dependent effects have also been reported (2, 3, 6), deemed to be associated with FTY720's metabolic burden on the liver (7). Other dose-dependent unwanted and toxic effects of FTY720 due to S1PR agonism, e.g., macular edema (8), unwanted loss of weight and appetite (9), etc. have been reported.

FTY720 itself was derived from myriocin (ISP-1) – a secondary metabolite of the entomopathogenic fungus *Isaria sinclairii* and a potent inhibitor of the serine palmitoyltransferase (SPT) enzyme in the *de-novo* sphingolipid biosynthesis pathway (10). Myriocin alters a metabolic pathway in eukaryotic cells vital for their stability and proliferation. Consistently, it has shown to have antifungal effects e.g., against *Candida* and *Aspergillus spp.* (11, 12).

While FTY720 has no activity against SPT, emerging studies (13, 14) are pointing towards S1PR-independent anti-proliferative effects of FTY720 in its non-phosphorylated form. Meanwhile, several cases of opportunistic fungal infections are reported among people receiving FTY720 chronically (15, 16). Although these reports describe case studies and population-based evidence is lacking in this regard, it seems unclear whether FTY720 facilitates fungal expansion *in vivo* by weakening the immune system, or has retained the antifungal effect of its molecular ancestor.

The subject of identifying and confirming the possible antifungal properties of substances like FTY720 – which could be administered safely in humans – gains more relevance when considering the rapid emergence of serious mycosis cases resistant to our usual antifungal armamentarium. Among the candidiasis cases, resistance to drugs is classically attributed to the less prevalent infections with non-

albicans species, however, recent studies are documenting an alarming increase in the antifungal resistance of *Candida* ~~(C.)~~ *albicans* – the dominant pathogen in candidiasis cases (17, 18). As the incidence of opportunistic candidiasis is rising, and commensal *C. albicans* has a high prevalence (19), its resistance to drugs could be a major complicating factor of the future candidiasis cases.

Therefore, we first aimed to determine whether FTY720 has antifungal effects *in vitro* using antifungal susceptibility testing. Then, we aimed to characterize its effects on immunological responses and fungal burden values in mice infected with the opportunistic yeast *C. albicans* – one of the most prevalent causative agents of systemic opportunistic mycosis. We hereby report the study in accordance with the Animal Research: Reporting of In Vivo Experiments 2.0 (ARRIVE 2.0) guidelines (available at: https://arriveguidelines.org/).

2. Methods

2.1. Design

The present study was conducted from October 2019 until July 2020 in the Islamic Azad University of Falavarjan, Isfahan, Iran. It could be divided into an *in-vitro* substudy and an *in-vivo* substudy, with the former aiming to measure FTY720's minimum inhibitory concentration (MIC) for *C. albicans*, and the later aiming to characterize its effect on the immune response measures and fungal burden values in mouse candidiasis models. The later substudy (from now on referred to as the *in-vivo* study) was designed with the following formulation of research question:

- Population: female C57BL/6 mice.
- Interventions:
  - Oral FTY720
  - Intraperitoneal (IP) *C. albicans* inoculum
- Comparison:
  - Oral normal saline (oral placebo)
  - IP normal saline (injectable placebo)
- Outcomes and rationale of each:
  - Complete blood count with differential (CBC diff) and serum concentrations of interferon-gamma (IFNγ) and interleukin-10 (IL10) after 21 days of receiving oral FTY720/placebo: to characterize the immune responses of the mice.
  - Colony count of *C. albicans* in samples from the vaginas, kidneys, and livers of the mice after 21 days of receiving oral FTY720/placebo: to measure the fungal burden (20).

Based on the formulation of the research question, the *in-vivo* study was conducted with six arms (Fig.1):

1. Control: Six mice receiving oral and injectable placebo.
2. Only FTY720: Six mice receiving oral FTY720 and injectable placebo.

3. Only *C. albicans*: Six mice receiving oral placebo and IP *C. albicans* inoculum.
4. FTY720 after *C. albicans*: Ten mice receiving oral FTY720 from two days after IP *C. albicans* inoculum.
5. FTY720 with *C. albicans*: Ten mice receiving oral FTY720 and IP *C. albicans* inoculum on the same day.
6. FTY720 before *C. albicans*: Ten mice receiving oral FTY720 from two days before receiving IP *C. albicans* inoculum.

The only exclusion criterion for the *in-vivo* study was death due to an unrelated reason. No other eligibility criteria were considered.

[Figure 1 placeholder]

2.2. *In-vitro* antifungal susceptibility testing

2.2.1. Well diffusion assay

We first used a *qualit*[illegible]ion assay in order to i) estimate the prob[illegible]TY720's *in-vitro* MIC for *C. albicans* and ii) t[illegible] of the drug serial concentration [illegible]or the following microbroth dilution assay. For the well diffusion assay, a 0.5 McFarland standard inoculum of *C. albicans* (CBS2747, Tehran University, Iran) was spread on three plates of Sabouraud dextr[illegible](SDA) (Scharlau, Turkey). A total of eleven sample wells (four wells in two plates, and three[illegible] each with a diameter of six millimeters were created in the plates using a sterile Pasteur pipette. One well was filled with normal saline as control while the others were filled with 200 μL of FTY720 suspensions with serial concentrations from 0.005 to 1.5 mg/mL. The visual presence of a zone of inhibition around each well was determined after a 30-hour incubation at 37 °C. The lowest concentration for which a zone of inhibition surrounded the corresponding well was considered as the probable MIC.

2.2.2. Microbroth dilution assay

After obtaining the probable MIC using the well diffusion assay, for precise and standardized measurement the MIC, a microbroth dilution assay – the gold-standard method for MIC measurement according to almost all standard laboratory guidelines – was performed following the methods described by the The European Committee on Antimicrobial Susceptibility Testing (EUCAST) (21). Briefly, microtubes in a sterile microtube plate were filled with 10 μL of a 0.5 McFarland standard inoculum of *C. albicans*, 90 μL of Sabouraud dextrose broth (SDB) (Scharlau, Turkey), and 100 μL of serially-diluted suspensions of FTY720, from 0.0005 to 0.5 mg/mL (i.e., the microtubes finally contained FTY720 suspensions with serial dilutions from 0.00025 to 0.25 mg/mL). One microtube was added by normal saline instead of FTY720 (positive control), and one was filled with sterile SDB without the *C. albicans* inoculum (negative control). The optical density (OD) of each microtube was read and documented at 640 nm immediately after preparation, and after a

24-hour incubation at 37 °C. The minimum drug concentrations for which the post-incubation change in OD was <1% and <50% were considered as the MIC_{99} and the MIC_{50}, respectively.

2.3. Preparation, allocation, and follow-up of the mice

For the *in-vivo* study, 48 female C57BL/6 mice (Royan Institute, Iran) – each weighing 22 (±3) grams – were prepared in accordance with the National Research Council guidelines for laboratory animal care (22). All mice were kept for one week before randomization, in standard cages in a humidity-controlled room at a 25 °C (±2) temperature and a 12 hours daylight, 12 hours darkness cycle. Thereafter, each mouse was identification-marked by an ear tag, and allocated to each one of the study arms based on a random sequence generated by the NumPy software package (for Python version 2.7 on MacOS). They received their interventions in an open-label manner and were followed up daily for 21 days after starting oral FTY720/placebo. FTY720 (0.3 mg/kg/day) or oral placebo was administered by gastric gavage in form of a 100 μL suspension. For determination of frequency and dosage of FTY720, we selected a regimen frequently used in previous studies, proven to bear immunomodulatory effects, and associated with minimal adverse reactions in C57BL/6 mice (23-29). The mice assigned to be infected with *C. albicans* were intraperitoneally injected with an inoculum containing 1.5 x 10^5 colony forming units (CFU) of *C. albicans*, while the others were injected with normal saline in the same amounts. At the end of the follow-up, the mice were sedated with ketamine-xylazine (100-10 mg/kg) and 1 mL of blood was taken from each through cardiac catheterization. The blood samples were taken in anticoagulated tubes, and were promptly sent to be prepared for CBC diff and cytokine assays. Then, through sterile procedures, the mice's livers, kidneys, and vaginal samples were extracted and sent to be prepared for fungal culture. The mice were, thereafter, euthanized with anesthetic overdose per American Veterinary Medical Association (AVMA) guidelines (30).

2.4. Analyses of samples

In order to characterize the mice's immune responses, absolute WBC and lymphocyte counts, serum IL10 (an anti-inflammatory cytokine), and IFNγ (a proinflammatory cytokine) concentrations were measured. CBC diff was performed on whole blood samples by manual hemocytometry (Neubauer chamber) (31). IFNγ and IL10 concentrations were quantified using ELISA (BioAssay™ kit for mouse, USBiological, USA) in accordance with the manufacturer's instructions. For fungal culture, the vaginal samples were directly spread on SDA plates. The livers and kidneys were first homogenized and filtered (using sterile techniques and equipment), were diluted in ten serial concentrations, and then spread on SDA plates. After a 30-hour incubation at 37 °C, the number of CFU's were counted and documented.

2.5. Statistical methods

2.5.1. Data preparation and determination of appropriate tests

The distributions of immune response measures (IFNγ, IL10. WBC, and ALC) were assumed to be lognormal; these measures were $\log_{10}$-transformed for the analyses. The distribution of fungal burden

values was assumed to be normal. The normality/lognormality assumptions were tested using the Kolmogorov-Smirnov method (32, 33); if the assumptions were confirmed by alpha>0.05, parametric tests (e.g., one-way analysis of variance [ANOVA]), and if not, non-parametric tests (e.g., Kruskal-Wallis) were used for those measures. Post-hoc and correction for multiple comparisons was performed by controlling the false discovery rate, using the two-stage step-up method of Benjamini, Krieger, and Yakutielli (34).

In order to determine whether a single pooled variance could be used in the parametric comparisons, the group variances were compared using the Brown-Forsyth test (35). In cases of insignificant (P>0.05) difference among variances of the arms, a single pooled variance was calculated and used; otherwise, the Welch and Brown-Forsyth method (36) was used (variances were calculated for individual arms separately).

2.5.2. Analyses and their rationale

To confirm if the follow-up period was sufficient for FTY720 to implement its immunological effects, the IFNγ, IL10, WBC, and ALC measures were compared between the control arm (arm 1) and the arm receiving FTY720 with injectable placebo (arm 2).

To confirm if the follow-up period was sufficient for the mice to develop an immune response against the fungal infection, the IFNγ, IL10, WBC, and ALC measures in the control arm (arm 1) were compared with the arm receiving IP *C. albicans* inoculum with oral placebo (arm 3).

To confirm if the follow-up period was sufficient for *C. albicans* to disseminate *in vivo*, the fungal burden values in the control arm (arm 1) were compared with the arm receiving IP *C. albicans* inoculum with oral placebo (arm 3).

To characterize the effect of FTY720 on mice's immunological response to *C. albicans*, the IFNγ, IL10, WBC, and ALC measures in the arms 4,5,6 (i.e., the arms receiving both FTY720 and IP *C. albicans*) were once compared with the arm receiving FTY720 with injectable placebo (arm 2), and once with the arm receiving IP *C. albicans* with oral placebo (arm 3).

In order to characterize the effect of FTY720 on the fungal burden *in vivo,* the liver, kidney, and vaginal colony count values in arms 4,5,6 (i.e., the arms receiving both FTY720 and IP *C. albicans)* were compared with the arm receiving IP *C. albicans* with oral placebo (arm 3).

2.5.3. Software

The Prism software (version 9.0.2 for MacOS; GraphPad Software LLC.) was used for statistical analysis and graphing.

2.6. Reproducibility, safety and ethical considerations

In order to ensure the reproducibility of the results, the Enhancing the QUAlity and Transparency Of health Research (EQUATOR) guidelines (37) were followed to report the study, enabling all researchers across the globe to repeat the experiments, obtain, compare, and validate the results.

The laboratory researchers followed strict regulatory standards in terms of safety; microbiological Petri dishes were not handled without gloves during any of the experiments, and the laboratory researchers wore sterile gowns, protective face-shields and masks, as well as following standard hand-washing protocols with povidone iodine solution before entering and after exiting the laboratory. All of the safety procedures were approved by the regulatory scientific review board who were monitoring the experiments through their surveillance systems.

The animal subjects of this study were kept in standard cages and were not restricted in terms of space, water, and food. All procedures on animal subjects were executed in accordance with national ethical guidelines. This study was approved by the ethics committee of the Islamic Azad University Falavarjan branch (Approval ID: IR.IAU.FALA.REC.1401.010).

3. Results

3.1. In-vitro antifungal susceptibility tests

In the well diffusion assay, the lowest concentration of FTY720 for which a visible zone of inhibition surrounded its corresponding well was 0.5 mg/mL (Fig.2), hence, the probable MIC of FTY720 was estimated to be 0.5 mg/mL or lower. As mentioned, we then used the resulted qualitative measures for precise and standard MIC measurement with a microbroth dilution assay, in which the MIC_{99} was measured to be 0.25 mg/mL, and the MIC_{50} was measured to be 0.12 mg/mL (Fig.3). These results confirmed that FTY720 comprises antifungal effects *in vitro*.

[Figure 2 placeholder]

[Figure 3 placeholder]

3.2. Overview and validation of the in-vivo study

The study was conducted per protocol; no unintended events happened during the study. The mice were randomized and allocated to the study arms two days before receiving their oral interventions (T -2). All of the randomized mice completed were followed up for 23 days (until T +21), except for one mouse in the control arm (arm 1), which died due to an unknown reason in the second day of oral normal saline consumption (T +2, four days after randomization). An investigative dissection of the mouse was conducted to identify the possible reason of death, but no gross abnormalities were detected. Samples from the normal saline administered to the mouse was cultured, resulting in growth of no microorganisms after one week. Apart from the signs of sepsis in the mice receiving IP *C. albicans* inoculum, the other mice experienced no serious adverse events during their follow-up.

At the end of the study, the immunological profile of the mice treated with FTY720 and injectable placebo (arm 2) significantly differed from the controls (arm 1) (Fig.4a), indicating a sufficient follow-up period for

FTY720 to execute its immunomodulatory effects. FTY720 significantly decreased IFNγ, increased IL10, and decreased white blood cell (WBC) and absolute lymphocyte (AL) counts (Fig.4a).

The *C. albicans*-infected mice receiving oral placebo (arm 3) showed significantly different immunological profiles and fungal burden measures with the controls (arm 1) (Fig.4a); this indicated that the follow-up period was sufficient for dissemination of *C. albicans in vivo,* and for an antifungal immunological response to develop. Disseminated candidiasis significantly increased IFNγ, WBC, and AL counts but insignificantly affected IL10 (Fig.4a). Cultures of liver, kidney, and vaginal samples of the mice not receiving IP *C. albicans* showed no growth of fungi, whereas the samples from all of the *C. albicans*-injected ones showed growth of *C. albicans* – confirmed with gross visual examination and direct smear microscopy (Fig.5, Fig.6). In two cultures (one kidney sample from a mouse in arm 3 and one vaginal sample from a mouse in arm 6) multiple microorganisms were colonized, highly indicative of procedural contamination; they were excluded from analyses.

[Figure 4 placeholder]

[Figure 5 placeholder]

[Figure 6 placeholder]

3.3. Effect of FTY720 on mice's immune response to C. albicans

In order to characterize the effect of FTY720 on the mice's immune response to *C. albicans*, we compared the arms receiving the IP *C. albicans* inoculum before, after, or simultaneously with initiation of FTY720 (arms 4,5,6), with the ones receiving FTY720/injectable placebo or *C. albicans*/oral placebo (arms 2,3) (Fig.1).

Similar to the mice receiving IP *C. albicans*/oral placebo (arm 3), *C. albicans* inoculation before, and simultaneously with initiation of FTY720 (arms 4,5) significantly increased IFNγ levels compared to the mice receiving FTY720/IP placebo (arm 2) (Fig.4b) (eTable 1). However, *C. albicans* inoculation after two days of FTY720 consumption (in arm 6) caused an insignificant increase in IFNγ levels; IFNγ levels were significantly lower in these mice compared to the mice receiving *C. albicans*/oral placebo (arm 3) (Fig.4b) (eTable 1). In simple words, being on FTY720 was required for at least two days at the time of candidiasis induction, for it to hinder the IFNγ response of the mice to the disseminating fungal infection.

The mice infected before initiation of FTY720 (arm 4) had significantly lower levels of IL10 compared to the ones receiving FTY720/IP placebo (arm 2), while being comparable to the ones receiving IP *C. albicans*/oral placebo (arm3) (Fig.4b) (eTable 1). It could be interpreted that the prior fungal infection hindered the IL10-increasing effect of FTY720. Induction of candidiasis simultaneously with, or later than FTY720 initiation (in arms 5,6) did not affect the IL10-increasing effect of FTY720 as shown in Figure 4b.

As interpreted before, FTY720 significantly decreased, and candidiasis significantly increased WBC and AL counts in mice (Fig.4a). The mice which started taking FTY720 before or after candidiasis induction

(arms 4,6) showed significantly lower WBC and AL counts than the ones receiving IP *C. albicans*/oral placebo (arm 2) (Fig.4b). Surprisingly, the mice with simultaneous initiation of FTY720 and induction of candidiasis still showed significantly increased WBC and AL counts – significantly higher than the mice receiving FTY720/injectable placebo (arm 2), and comparable to the ones receiving *C. albicans*/oral placebo (arm 3) (Fig.4b) (eTable 1).

3.4. Effect of FTY720 on fungal burden in vivo

To demonstrate the antifungal effect of FTY720 *in vivo*, we cultured specimens of mice's livers, kidneys, and vaginas 21 days after initiation of FTY720/oral placebo, and compared the resulted fungal burden values between the mice receiving *C. albicans*/oral placebo (arm 3), and the ones receiving both FTY720 and IP *C. albicans* inoculum (arms 4,5,6). The mice receiving FTY720 before, after, or simultaneously with induction of candidiasis showed significantly lower fungal burden measures in their livers and kidneys (eTable 1) (Fig.7); interestingly, the ones initiating FTY720 and getting infected on the same day (arm 5) – which also showed the high AL counts before – had the lowest liver/kidney fungal burdens – significantly lower than all of the other arms. Regarding the vaginal burdens, only the mice initiating FTY720 before receiving the IP *C. albicans* inoculum (arm 6) showed lower values than the ones receiving IP *C. albicans*/oral placebo (arm 3) (eTable 1) (Fig.7).

[Figure 7 placeholder]

4. Discussion

Our results demonstrated that FTY720 has antifungal effects against *C. albicans* – both *in vitro* and *in vivo*. In the mice models of disseminated candidiasis, this effect varied based on the chronological relation of FTY720 initiation and *C. albicans* inoculation. Our report is the first describing the *in-vivo* antifungal effect of FTY720 as far as we know.

In line with our study, a recent *ex-vivo* study by Wei and colleagues pointed to the fungicidal effect of FTY720, which synergized the effect of amphotericin B (38). Wei et al. showed that this fungicidal effect is present against non-albicans Candida spp., *Saccharomyces cerevisiae*, and *Cryptococcus neoformans* as well as *C. albicans* (38). They also showed that 0.011 mg/mL of FTY720 affects *C. albicans'* growth rate similar to 0.08 mg/L of amphotericin B (38). In an attempt to characterize the cause of this effect, they demonstrated that FTY720 induces production and hyperaccumulation of reactive oxygen species (ROS) in a dose-dependent manner; scavenging of the ROS from the samples using N-acetyl cysteine (NAC) compromised the fungicidal effect of FTY720 significantly. Therefore, Wei et al.'s study suggested hyperaccumulation of ROS as the reason of FTY720's fungicidal effect (38). ROS accumulation in fungi induced by unphosphorylated FTY720 has been observed in previous studies as well (14, 39), the mechanism of which is an area of active investigation.

Administration of FTY720 at 1.25 mg/day in healthy adults – above which has proved unbeneficial for people with MS (6) – results in a maximum blood concentration (C_{max}) of 10.2 (±2.7) ng/mL at steady-state (7); this concentration is 25000 times lower than its MIC_{99} for *C. albicans.* Maximum concentrations of FTY720 in kidney and liver tissues reach approximately 40 times the C_{max} (40) – still much less than its *in-vitro* MIC_{99}. Therefore, the current FTY720 regimens in people with MS are doubted to directly restrict *C. albicans* infection. In our *in-vivo* study, the mice received 0.3 mg/kg/day of FTY720. A single 0.3 mg/kg dose of FTY720 is associated with a maximum liver and kidney concentration of around 1.5 µg/mL (40). Considering a 10-fold steady-state concentration reached by daily administration, the maximum concentration of FTY720 in liver and kidney reaches 15 µg/mL – 17 times less than its *in-vitro* MIC_{99} and 8 times less than its MIC_{50}. Despite this fact, we did observe the antifungal effect of FTY720 *in vivo*. Hence, we speculate that FTY720 had a synergistic effect with host's innate immunity against *C. albicans*. The yeast could antagonize oxidative stress up to a specific threshold by inducing antioxidant metabolic pathways (41). The innate immunity is absent *in vitro*, therefore, higher concentrations of FTY720 are needed to raise the ROS levels up to the threshold intolerable by the yeast. *In vivo*, the generated ROS induced by FTY720, although may not be adequate by itself, is added by the ROS produced by the innate immune cells, while also attracting more cells to the frontline (42), facilitating their phagocytizing, and easing the killing of fungi by them (38). Nevertheless, countless mechanistic factors are involved in fungal proliferation and expansion *in vivo*; determination of our observations' mechanistic background could be an interesting subject for future studies.

As mentioned in the introduction, some case studies focusing on atypical cryptococcal infections (15, 16) hypothesize that people with MS receiving FTY720 may be generally more susceptible to fungal infections. However, Wei et al. demonstrated that the *ex-vivo* fungicidal effect of FTY720 against *Cryptococcus neoformans* is similar to its effect on *C. albicans,* and we demonstrated that the *in-vitro* antifungal effect of FTY720 against *C. albicans* is also present *in vivo.* The dose-dependency of FTY720's antifungal effect may be the explanation behind this discrepancy. It could be hypothesized that FTY720's immunosuppressive effect when administered chronically with low doses – as in people with MS – facilitates fungal infections, as its antifungal effects are absent in concentrations reached with those regimens (discussed above).

Another notable observation in our study was the mice with simultaneous C. albicans inoculation and initiation of FTY720 (arm 5) had a prominent increase in AL counts, similar to the mice receiving *C. albicans*/oral placebo (arm 3); however, their kidney and liver samples revealed significantly lower fungal burdens. The decreased fungal burden values in these mice could not merely be explained by the increased AL counts, otherwise, the mice receiving *C. albicans*/oral placebo (arm 3) who had similar AL counts would have similarly shown low fungal burdens in their organs. Further fundamental work is warranted to explain this interesting observation.

5. Conclusion

We showed that the immunomodulatory drug FTY720 has an antifungal effect *in vitro*, which is present at even lower concentrations *in vivo*. The facilitation of ROS hyperaccumulation by FTY720 – which assists the innate immune system in creating an antifungal environment – may explain our observations. Future studies are warranted to evaluate further aspects of repositioning FTY720 for antifungal use in clinical settings.

6. Limitations

In order to ensure a sterile and prompt procedure of homogenization and fungal culturing, we regret that we did not perform any sectioning, staining, and direct microscopy of the liver, kidney, and vagina samples to provide the visual confirmation of *C. albicans* colonization *within* the tissues of the mice in treated and control groups. Also, mainly due to limited resources, the *in-vitro* effect of FTY720 on important virulence traits of *C. albicans,* including filamentation, adhesion, and biofilm formation was not investigated in any standardized manner. Furthermore, the photographic documentations of the experiments provided in this report may not be considered in accordance with research standards; therefore, providing research-grade photographic documentations of the experiments conducted in our study and their visual validation future studies is warranted to contribute to the reproducibility of the results. Other limitations included outcome measurement at a single timepoint rather than several timepoints during the study, usage of limited number of assays, relatively short follow-up period, open-label fashion, not measuring drug concentrations in blood and tissues after administration, not measuring the ROS concentrations *in vitro* or *in vivo*, and other possible unnoticed biases. Further replicative studies are encouraged to add to our knowledge and account for the limitations of our study.

7. Conflict of interest and funding

None.

8. Author contributions

NN: conceptualization; methodology; investigation; data curation; resources; writing – original draft; validation; project administration.

MM: supervision; validation.

ME: supervision; writing – review and editing.

NS: methodology; formal analysis; visualization; writing – original draft; writing – review and editing.

9. Acknowledgements

None.

10. Data availability

All data produced in this study are presented within the manuscript, its figures, and supplemental contents. Raw data is also available upon reasonable request from the corresponding author.

11. References

1. Roy R, Alotaibi AA, Freedman MS. Sphingosine 1-Phosphate Receptor Modulators for Multiple Sclerosis. CNS Drugs. 2021;35(4):385-402.
2. Kappos L, Radue E-W, O'Connor P, Polman C, Hohlfeld R, Calabresi P, et al. A placebo-controlled trial of oral fingolimod in relapsing multiple sclerosis. New England Journal of Medicine. 2010;362(5):387-401.
3. La Mantia L, Tramacere I, Firwana B, Pacchetti I, Palumbo R, Filippini G. Fingolimod for relapsing-remitting multiple sclerosis. Cochrane Database Syst Rev. 2016;4:Cd009371.
4. Wu X, Xue T, Wang Z, Chen Z, Zhang X, Zhang W, et al. Different Doses of Fingolimod in Relapsing-Remitting Multiple Sclerosis: A Systematic Review and Meta-Analysis of Randomized Controlled Trials. Front Pharmacol. 2021;12:621856.
5. Vargas WS, Perumal JS. Fingolimod and cardiac risk: latest findings and clinical implications. Ther Adv Drug Saf. 2013;4(3):119-24.
6. Wu X, Xue T, Wang Z, Chen Z, Zhang X, Zhang W, et al. Different doses of fingolimod in relapsing-remitting multiple sclerosis: a systematic review and meta-analysis of randomized controlled trials. Frontiers in pharmacology. 2021;12:1132.
7. David OJ, Kovarik JM, Schmouder RL. Clinical pharmacokinetics of fingolimod. Clinical pharmacokinetics. 2012;51(1):15-28.
8. Cugati S, Chen CS, Lake S, Lee AW. Fingolimod and macular edema: Pathophysiology, diagnosis, and management. Neurol Clin Pract. 2014;4(5):402-9.
9. Etemadifar M, Khorvash R, Zolfagharifard H, Sedaghat N, Nouri H, Salari M. Weight Loss and Loss of Appetite as a Side Effect of Fingolimod. 2022.
10. Chiba K, Adachi K. Discovery of fingolimod, the sphingosine 1-phosphate receptor modulator and its application for the therapy of multiple sclerosis. Future Med Chem. 2012;4(6):771-81.
11. Perdoni F, Signorelli P, Cirasola D, Caretti A, Galimberti V, Biggiogera M, et al. Antifungal activity of Myriocin on clinically relevant Aspergillus fumigatus strains producing biofilm. Bmc Microbiology. 2015;15(1):1-8.
12. Yang X, Pei Z, Hu R, Zhang Z, Lou Z, Sun X. Study on the Inhibitory Activity and Possible Mechanism of Myriocin on Clinically Relevant Drug-Resistant Candida albicans and Its Biofilms. Biol Pharm Bull. 2021;44(3):305-15.
13. Zhang L, Wang H-D, Ji X-J, Cong Z-X, Zhu J-H, Zhou Y. FTY720 for cancer therapy. Oncology reports. 2013;30(6):2571-8.
14. Takasaki T, Hagihara K, Satoh R, Sugiura R. More than just an immunosuppressant: the emerging role of FTY720 as a novel inducer of ROS and apoptosis. Oxidative medicine and cellular longevity. 2018;2018.
15. Carpenter AF, Goodwin SJ, Bornstein PF, Larson AJ, Markus CK. Cutaneous cryptococcosis in a patient taking fingolimod for multiple sclerosis: here come the opportunistic infections? Multiple Sclerosis Journal. 2017;23(2):297-9.
16. Del Poeta M, Ward BJ, Greenberg B, Hemmer B, Cree BA, Komatireddy S, et al. Cryptococcal Meningitis Reported With Fingolimod Treatment: Case Series. Neurology-Neuroimmunology Neuroinflammation. 2022;9(3).

17. Whaley SG, Berkow EL, Rybak JM, Nishimoto AT, Barker KS, Rogers PD. Azole antifungal resistance in Candida albicans and emerging non-albicans Candida species. Frontiers in microbiology. 2017;7:2173.
18. Coste AT, Kritikos A, Li J, Khanna N, Goldenberger D, Garzoni C, et al. Emerging echinocandin-resistant Candida albicans and glabrata in Switzerland. Infection. 2020;48(5):761-6.
19. Romo JA, Kumamoto CA. On Commensalism of Candida. J Fungi (Basel). 2020;6(1).
20. Conti HR, Huppler AR, Whibley N, Gaffen SL. Animal models for candidiasis. Curr Protoc Immunol. 2014;105:19.6.1-.6.7.
21. EUCAST DEFINITIVE DOCUMENT E.DEF 7.3.1 Method for the determination of broth dilution minimum inhibitory concentrations of antifungal agents for yeasts. European Committee for Antimicrobial Susceptibility Testing (EUCAST). 2017.
22. Council NR. Guide for the care and use of laboratory animals. 2010.
23. Abdullah CS, Li Z, Wang X, Jin ZQ. Depletion of T lymphocytes ameliorates cardiac fibrosis in streptozotocin-induced diabetic cardiomyopathy. Int Immunopharmacol. 2016;39:251-64.
24. Bonfiglio T, Olivero G, Merega E, Di Prisco S, Padolecchia C, Grilli M, et al. Prophylactic versus Therapeutic Fingolimod: Restoration of Presynaptic Defects in Mice Suffering from Experimental Autoimmune Encephalomyelitis. PLoS One. 2017;12(1):e0170825.
25. Cheng Q, Li D, Liang H, Yang H, Lei D, Gao D, et al. Effects of long-term administration of low-dose FTY720 on survival of murine cardiac allograft. J Huazhong Univ Sci Technolog Med Sci. 2012;32(2):199-204.
26. Efstathopoulos P, Kourgiantaki A, Karali K, Sidiropoulou K, Margioris AN, Gravanis A, et al. Fingolimod induces neurogenesis in adult mouse hippocampus and improves contextual fear memory. Transl Psychiatry. 2015;5(11):e685.
27. Hwang MW, Matsumori A, Furukawa Y, Ono K, Okada M, Iwasaki A, et al. FTY720, a new immunosuppressant, promotes long-term graft survival and inhibits the progression of graft coronary artery disease in a murine model of cardiac transplantation. Circulation. 1999;100(12):1322-9.
28. Pinschewer DD, Ochsenbein AF, Odermatt B, Brinkmann V, Hengartner H, Zinkernagel RM. FTY720 immunosuppression impairs effector T cell peripheral homing without affecting induction, expansion, and memory. The Journal of Immunology. 2000;164(11):5761-70.
29. Wang G, Kim RY, Imhof I, Honbo N, Luk FS, Li K, et al. The immunosuppressant FTY720 prolongs survival in a mouse model of diet-induced coronary atherosclerosis and myocardial infarction. J Cardiovasc Pharmacol. 2014;63(2):132-43.
30. Underwood W, Anthony R. AVMA guidelines for the euthanasia of animals: 2020 edition. Retrieved on March. 2020;2013(30):2020-1.
31. Database. JSE. Basic Methods in Cellular and Molecular Biology. Using a Hemacytometer to Count Cells. JoVE, Cambridge, MA,. 2020.
32. Smirnov N. Table for Estimating the Goodness of Fit of Empirical Distributions. The Annals of Mathematical Statistics. 1948;19(2):279-81, 3.
33. Kolmogorov A. Sulla determinazione empirica di una lgge di distribuzione. Inst Ital Attuari, Giorn. 1933;4:83-91.
34. Benjamini Y, Krieger AM, Yekutieli D. Adaptive linear step-up procedures that control the false discovery rate. Biometrika. 2006;93(3):491-507.
35. Brown MB, Forsythe AB. Robust tests for the equality of variances. Journal of the American statistical association. 1974;69(346):364-7.
36. WELCH BL. THE GENERALIZATION OF ‘STUDENT'S’ PROBLEM WHEN SEVERAL DIFFERENT POPULATION VARLANCES ARE INVOLVED. Biometrika. 1947;34(1-2):28-35.
37. Groves T. Enhancing the quality and transparency of health research. British Medical Journal Publishing Group; 2008.
38. Wei L-Q, Tan J-C, Wang Y, Mei Y-K, Xue J-Y, Tian L, et al. Fingolimod potentiates the antifungal activity of amphotericin B. Frontiers in cellular and infection microbiology. 2021;11:346.
39. Hagihara K, Mizukura A, Kitai Y, Yao M, Ishida K, Kita A, et al. FTY 720 stimulated ROS generation and the S ty1/A tf1 signaling pathway in the fission yeast S chizosaccharomyces pombe. Genes to Cells. 2014;19(4):325-37.
40. Meno-Tetang GM, Li H, Mis S, Pyszczynski N, Heining P, Lowe P, et al. Physiologically based pharmacokinetic modeling of FTY720 (2-amino-2 [2-(-4-octylphenyl) ethyl] propane-1, 3-diol hydrochloride) in rats after oral and intravenous doses. Drug metabolism and disposition. 2006;34(9):1480-7.

41. Garcerá A, Casas C, Herrero E. Expression of Candida albicans glutathione transferases is induced inside phagocytes and upon diverse environmental stresses. FEMS yeast research. 2010;10(4):422-31.
42. Brothers KM, Gratacap RL, Barker SE, Newman ZR, Norum A, Wheeler RT. NADPH oxidase-driven phagocyte recruitment controls Candida albicans filamentous growth and prevents mortality. PLoS pathogens. 2013;9(10):e1003634.

Figure legends

Figure 1: Schematic overview of the *in-vivo* study arms.

Figure 2: Photographs of well diffusion plates after incubation. The wells 10 to 1 contained FTY720 with serial concentrations from 0.005 to 1.5 mg/mL respectively. The control "cont" well contained normal saline. Zones of inhibition are visible surrounding the wells 4 to 1, corresponding to serial concentrations from 0.5 to 1.5 mg/mL.

Figure 3: Results of the microbroth dilution assay for determining FTY720's MIC for *C. albicans*. The measured plotted on the Y-axis are resulted from subtracting the pre-incubation (background) from the post-incubation OD's. The hyphenated line corresponds to the best-fit regression model. Abbreviations: OD, optical density.

Figure 4: Immune profile of the mice at the end of the *in-vivo* study stratified by study arms. **(a)** comparison of immune response measures between the control mice (arm 1), the mice receiving FTY720/injectable placebo (arm 2), and the ones receiving IP *C. albicans* inoculum/oral placebo (arm 3). These measures confirm that the follow-up duration of the *in-vivo* study was sufficient. **(b)** comparison of immune response measures between the mice receiving FTY720/injectable placebo (arm 2), and the mice receiving IP *C. albicans* inoculum/oral placebo (arm 3), and the treated infected mice (arms 4,5,6); only comparisons with P<0.05 are shown. ns: P>0.05; *P<0.05; **0.05<P<0.01; ***0.01<P<0.001; ****P<0.0001. Abbreviations: IFNγ, interferon-gamma; IL10, interleukin-10; WBC, white blood cell; ALC, absolute lymphocyte count; IP, intraperitoneal; C. alb, *Candida albicans.*

Figure 5: Photographs of four selected culture plates *after* completion of the experiments and documentation of their results. It should be noted that *during* the experiments, none of the microbiological Petri dishes were handled without gloves as seen in these photographs. a) Vaginal sample from a mouse in arm 5; b) Liver sample from a mouse in arm 6; c) Vaginal sample from a mouse in arm 2 (above) and a mouse in arm 3 (below); d) Kidney sample from a mouse in arm 3.

Figure 6: Micrograph of gram-stained smears of colonies in vaginal sample cultures of two mice in arm 5. C. albicans yeasts are seen undergoing filamentation.

Figure 7: Colony counts of *C. albicans* in kidneys, livers, and vaginas of the infected arms at the end of the *in-vivo* study. ns: P>0.05; *P<0.05; **0.05<P<0.01; ***0.01<P<0.001; ****P<0.0001. Abbreviations: C. alb, *Candida albicans.*

Figure 1: Schematic overview of the in-vivo study arms.

Click here to access/download;Figure;Fig1.drawio.pdf

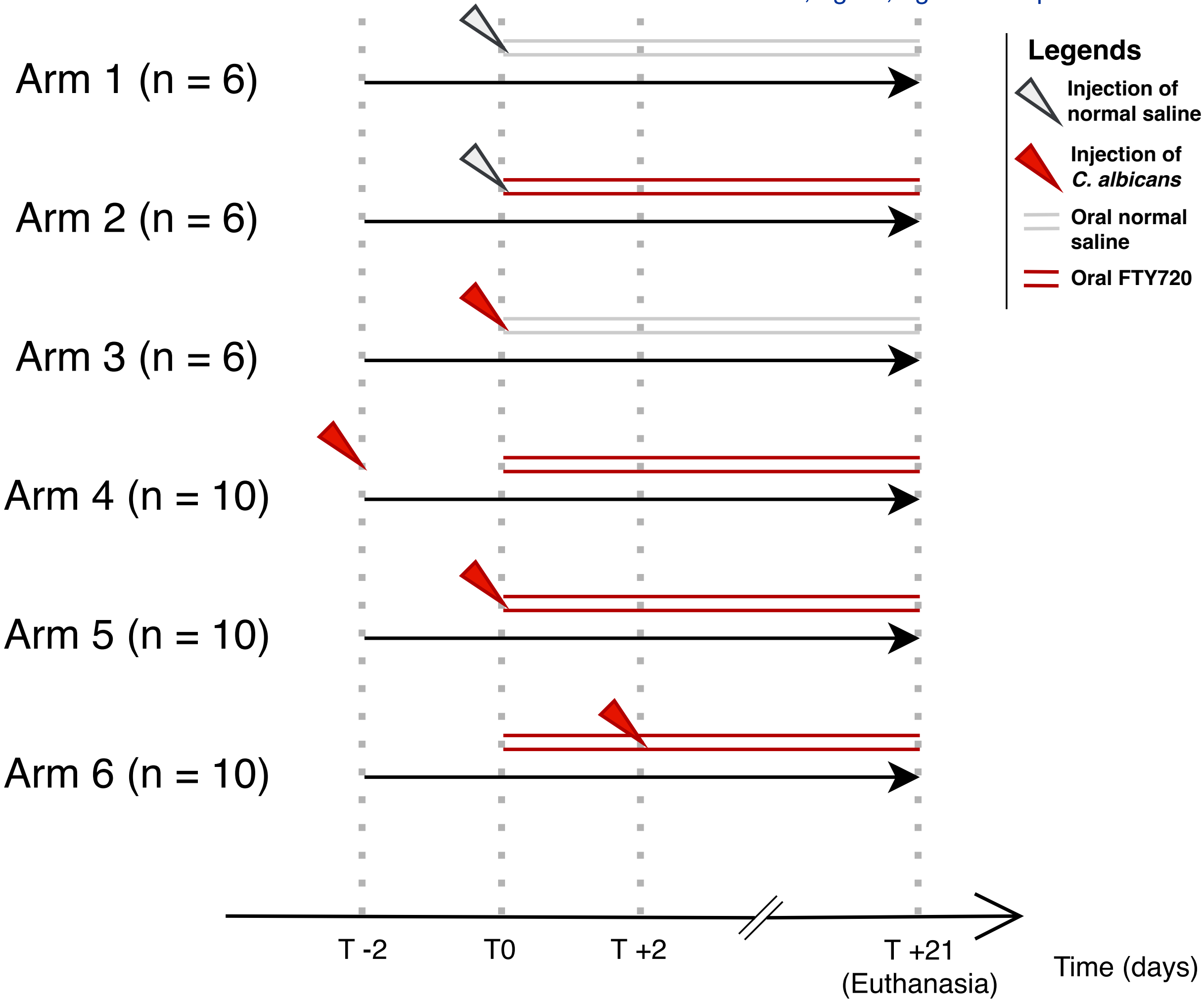

Click here to access/download;Figure;eFig1.pdf

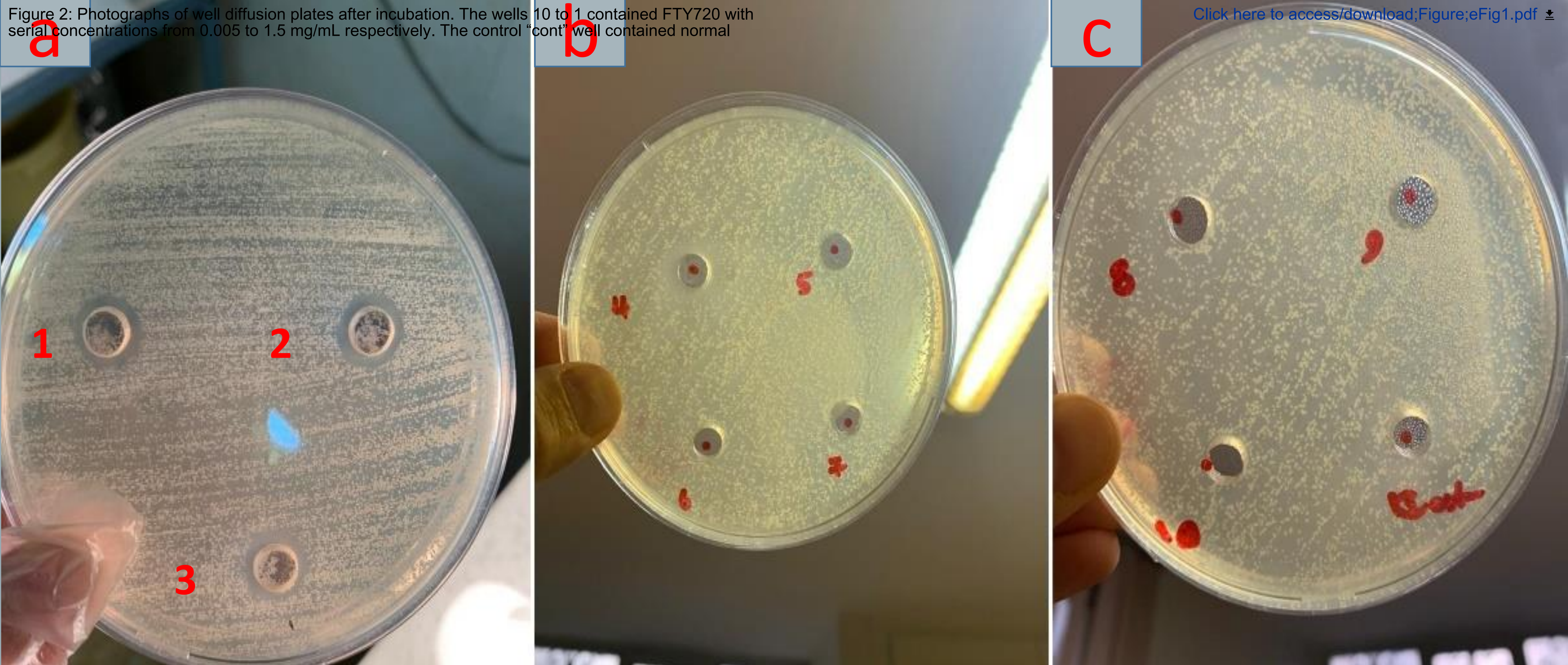

Figure 2: Photographs of well diffusion plates after incubation. The wells 10 to 1 contained FTY720 with serial concentrations from 0.005 to 1.5 mg/mL respectively. The control “cont” well contained normal

Figure 3: Results of the microbroth dilution assay for determining FTY720's MIC for C. albicans. The measured plotted on the Y-

Click here to access/download;Figure;Fig2.pdf

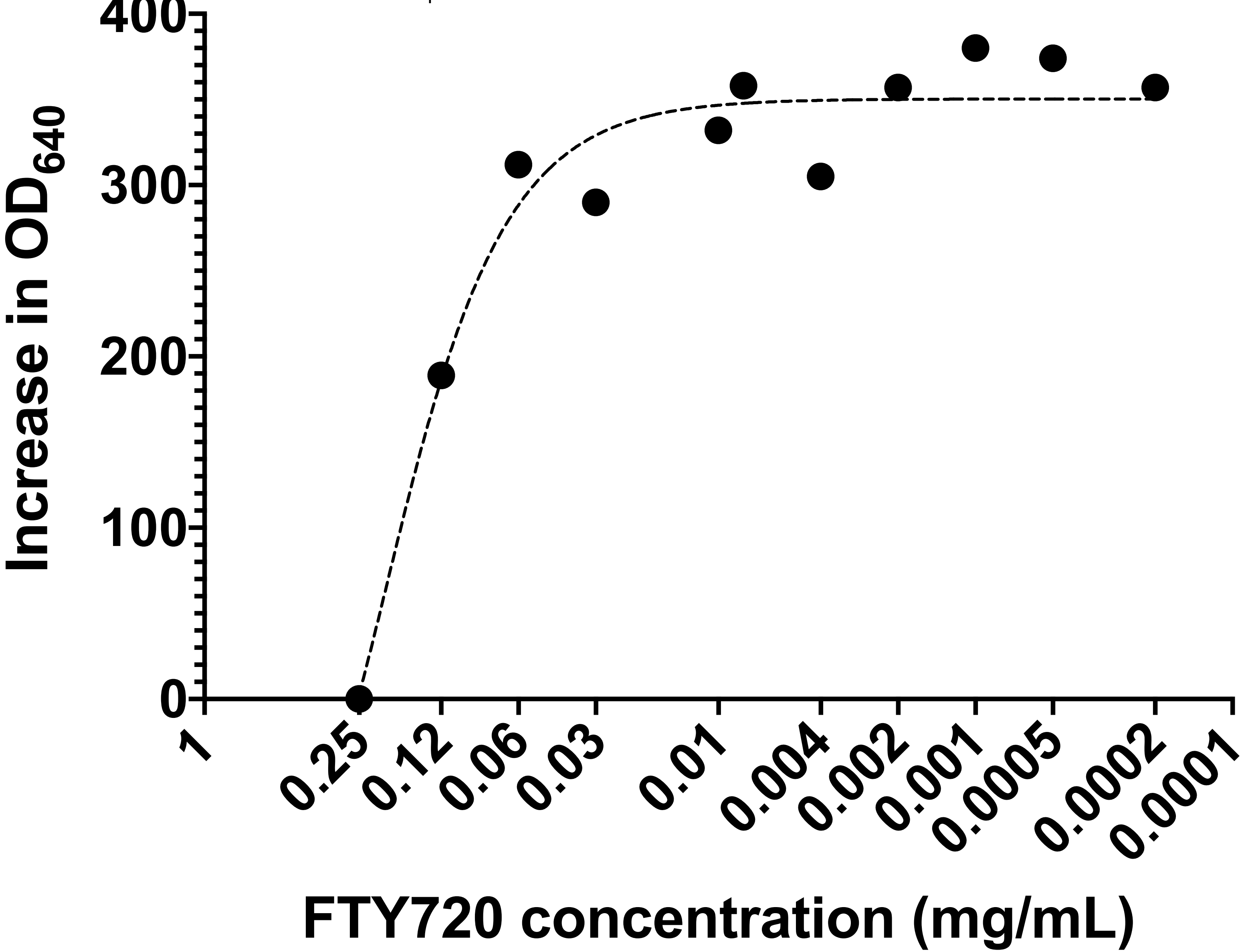

Figure 4: Immune profile of the mice at the end of the in-vivo study stratified by study arms. (a) comparison of immune response measures between the

Click here to access/download;Figure;Fig3 new new.pdf

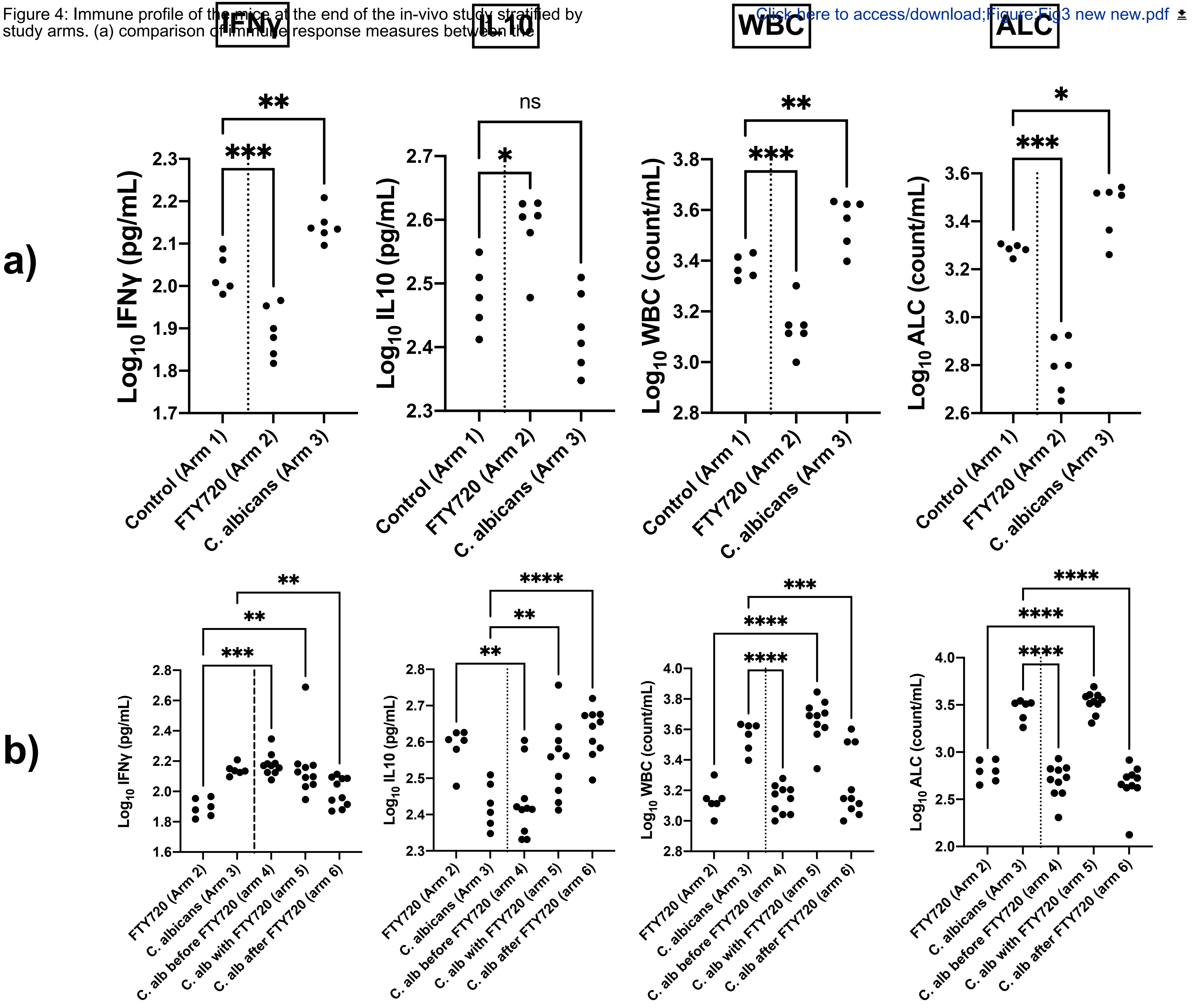

Figure 5: Photographs of four selected culture plates after completion of the experiments and documentation of their results.

Click here to access/download;Figure;eFig2.jpg

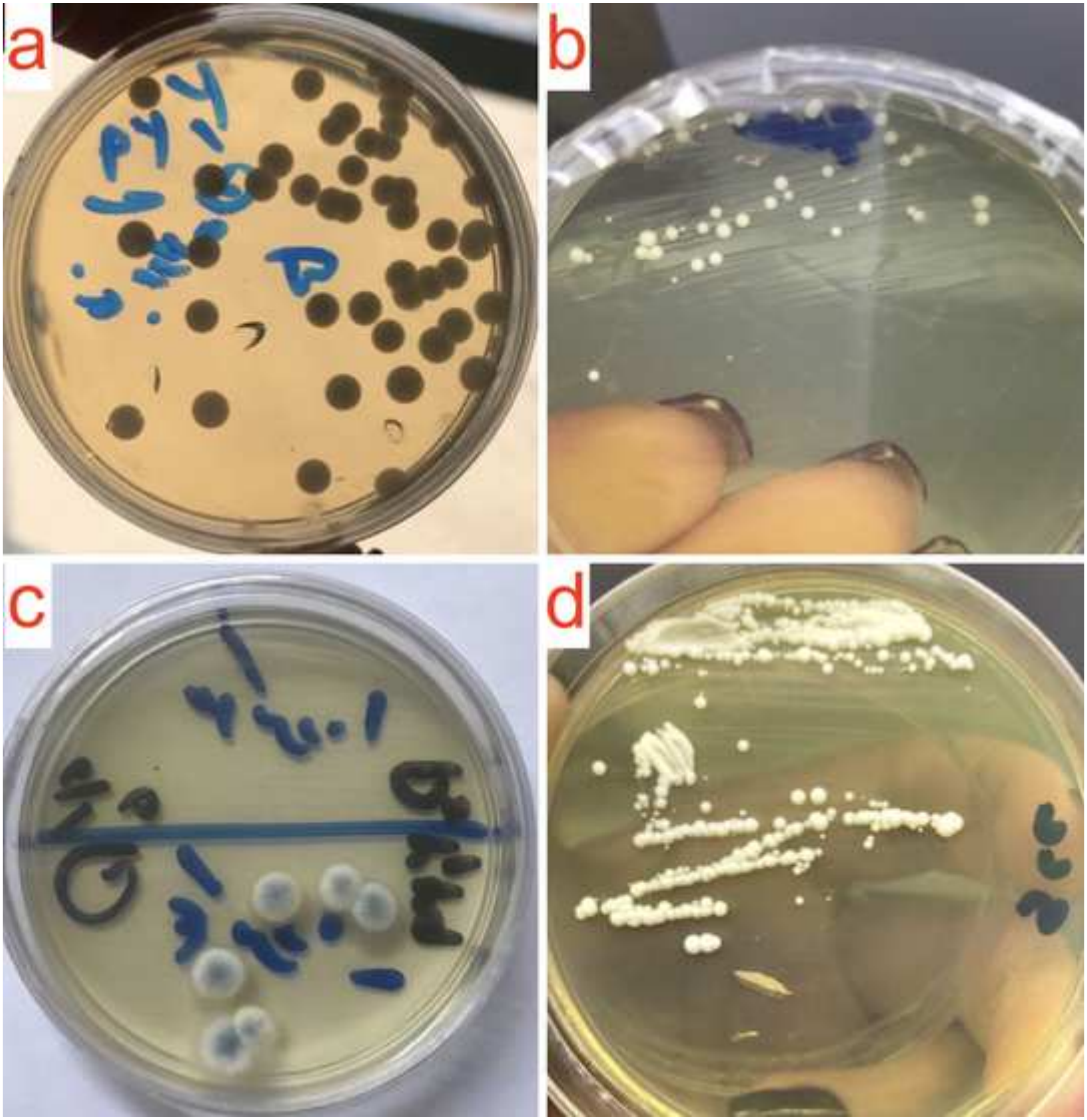

Figure 6: Micrograph of gram-stained smears of colonies in vaginal sample cultures of two mice in arm 5. C. albicans yeasts are seen undergoing filamentation.

Click here to access/download;Figure;eFig3.jpg

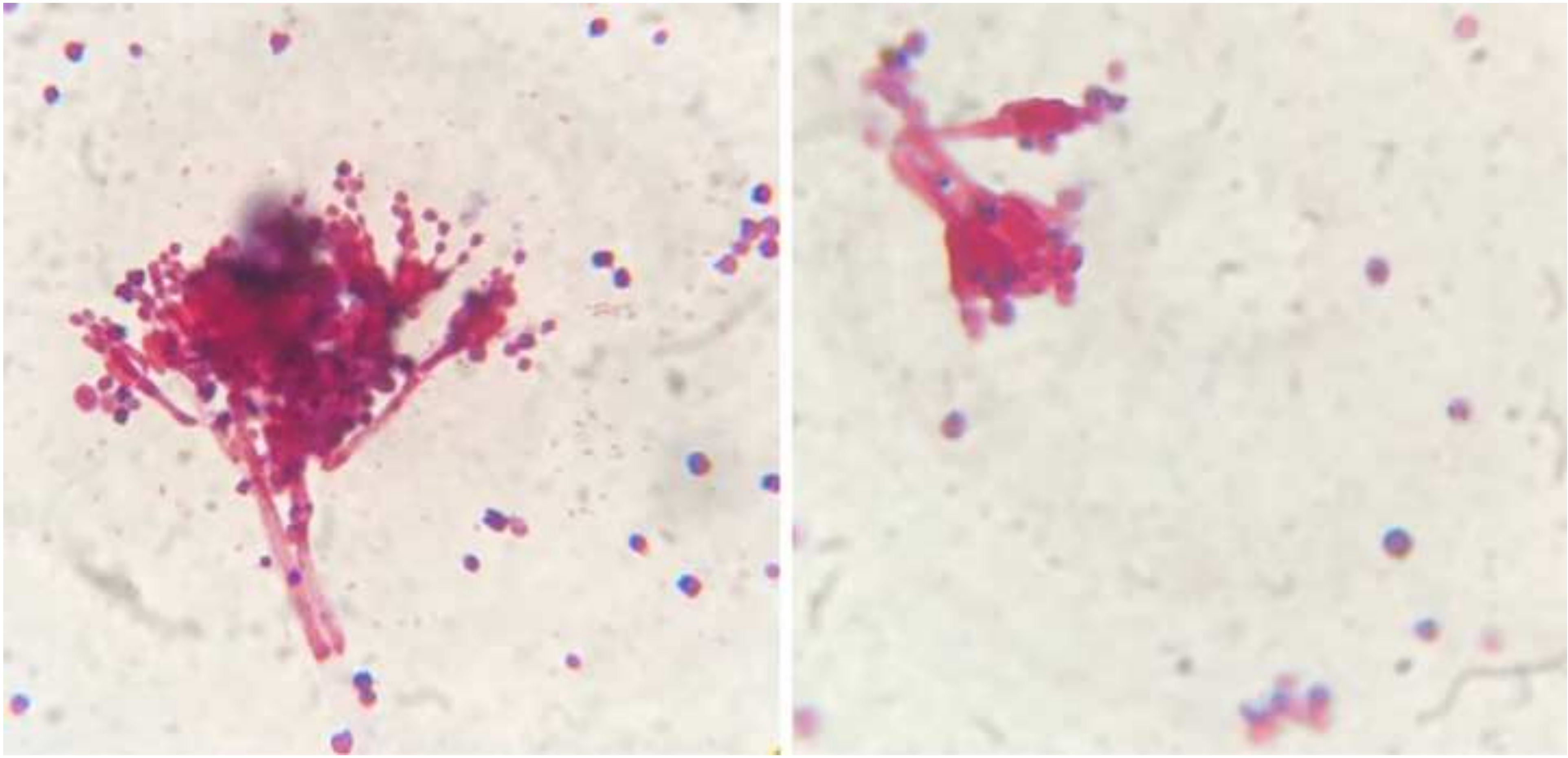

Figure 7: Colony counts of C. albicans in kidneys, livers, and vaginas of the infected arms at the end of the in-vivo study. ns: P>0.05;

Click here to access/download;Figure;Fig 4.pdf

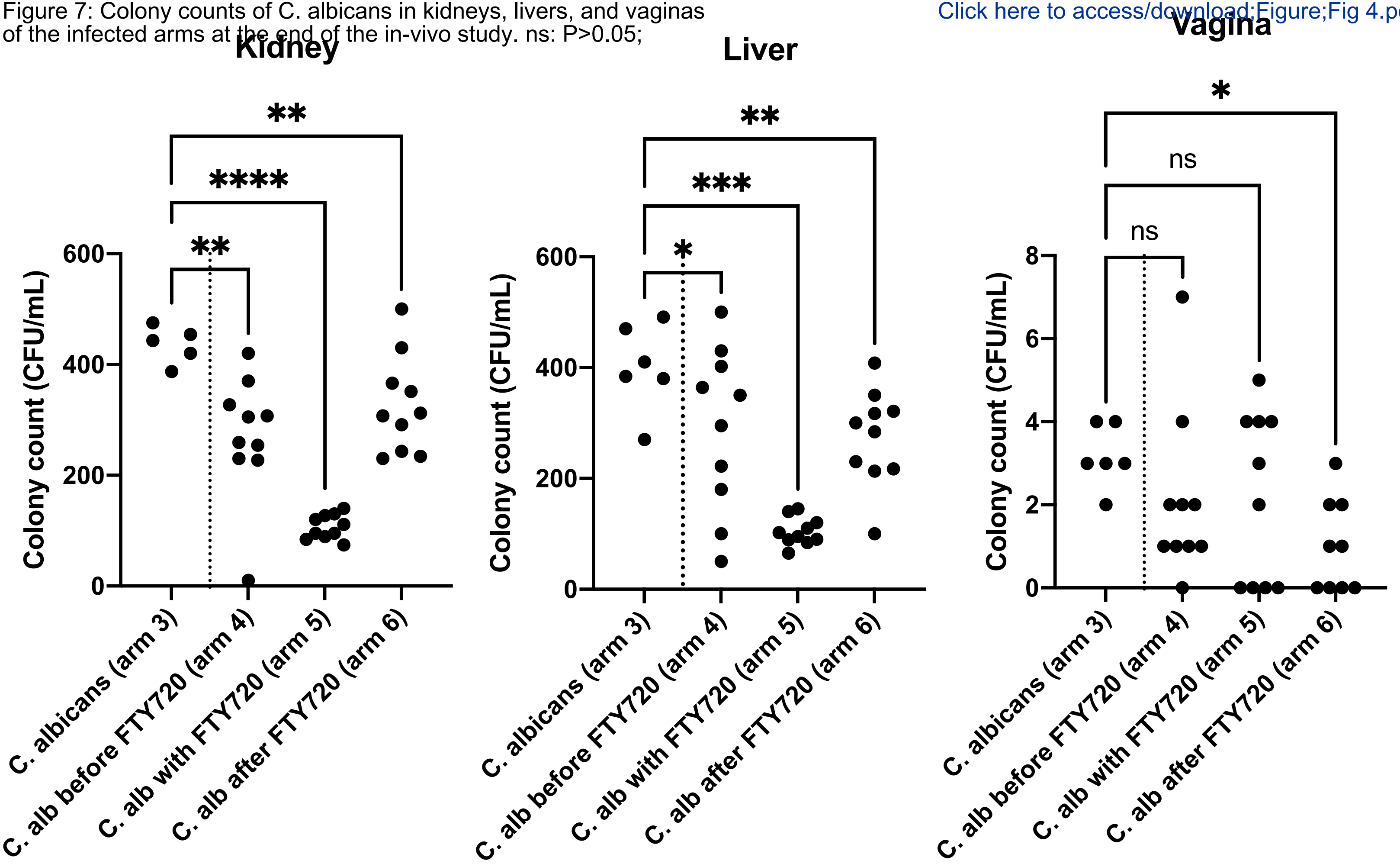

Click here to access/download
Supporting Information
Supp PONE.docx

Immunomodulatory drug fingolimod (FTY720) restricts the growth of opportunistic yeast *Candida albicans in vitro* and in a mouse candidiasis model

Short Title: Characterization of the antifungal effect of fingolimod (FTY720)

Niloofar Najarzadegan[a]; Mahboubeh Madani[a]; Masoud Etemadifar[b]; Nahad Sedaghat[b,c]*.*

a. *Department of Microbiology, Falavarjan Branch, Islamic Azad University, Isfahan, Iran.*
b. *Alzahra Research Institute, Alzahra University Hospital, Isfahan University of Medical Sciences, Isfahan, Iran.*
c. *Network of Immunity in Infection, Malignancy, and Autoimmunity (NIIMA), Universal Scientific, Education, and Research Network (USERN), Isfahan, Iran.*

**Address for Correspondence:*

Mahboubeh Madani

Department of Microbiology, Falavarjan Branch, Islamic Azad University, Isfahan, Iran.

Email: mmadani66@gmail.com

Nahad Sedaghat

Alzahra Research Institute, Alzahra University Hospital, Isfahan University of Medical Sciences, Isfahan, Iran.

Email: nahad.sedaghat@gmail.com

Manuscript details:

- Word count (main text): ~~3966~~4273
- Number of figures: 7~~4~~
- Number of tables: 0
- Number of supplemental files: 1
- Number of references: ~~35~~42

Abstract

Fingolimod (FTY720) is a drug derived from the fungicidal compound myriocin. As it was unclear whether FTY720 has antifungal effects as well, we aimed to characterize its effect on *Candida ~~(C.)~~ albicans in vitro* and in a mouse candidiasis model. In-vitro antifungal susceptibility testing was performed, along with a randomized, six-arm, parallel, open-label trial on 48 mice receiving oral FTY720 (0.3 mg/kg/day), intraperitoneal *Candida ~~C.~~ albicans* inoculum, or placebos with different combinations and chorological patterns. Immune response measures including interleukin-10, interferon-gamma, and absolute lymphocyte count, and fungal burden values including *Candida ~~C.~~ albicans* colony counts in liver, kidney, and vagina were measured as outcomes. Microbroth dilution assay revealed FTY720's minimum inhibitory concentration (MIC_{99}) to be 0.25 mg/mL for *Candida albicans*. Significant ($P<0.05$) reductions in fungal burden values of the infected mice receiving FTY720 were observed. As expected, the immune response measures were shifted towards an anti-inflammatory profile in the mice receiving FTY720. Since FTY720's MIC_{99} is not reached *in vivo* with the regimen we used, we hypothesize that FTY720 synergizes the host's innate immune functions by inducing the production of reactive oxygen species. Further studies are warranted to unveil the mechanistic explanations of our observations, and clarify further aspects of repurposing FTY720 for antifungal use in clinical settings.

Key words: Fingolimod; Candida albicans; candidiasis; Drug Repositioning.

Commented [MOU1]: Reviewer 1 comment 1
C. albicans was uniformly changed to candida albicans in the abstract.

Commented [MOU2]: Reviewer 1 comment 3
The required space between values and units were added.

Commented [MOU3]: Reviewer 1 comment 3
The required space between values and units were added.

1. Introduction

Fingolimod (FTY720) is an immunomodulatory drug currently indicated and approved by the FDA for treatment of the relapsing forms of multiple sclerosis (MS) – an autoimmune entity involving the CNS. It is used off-label for progressive MS and other autoimmune neuropathies, while being evaluated for extra-nervous pathologies as well (2-4). FTY720 is considered a prodrug of its phosphorylated form fingolimod-phosphate (FTY720-P). Being a modulator of the sphingosine-1-phosphate receptors (S1PR) by structural analogy, FTY720-P induces internalization of S1PR from the surface of lymphocytes, hindering their trafficking outside the primary lymphatics and, therefore, keeping them away from inflammatory sites in end organs ~~-~~(1).

While being associated with beneficial outcomes and reasonable tolerability among people with immune-mediated conditions such as MS (2, 3), safety and toxicity studies have shown both dose-dependent and time-dependent adverse effects associated with FTY720 (4). The time-dependent adverse effects have been opportunistic infections, reactivation of latent viruses, malignancies, etc., and were mostly deemed to be due to chronic immunosuppression (4). Among the dose-dependent adverse effects, the most common has been cardiotoxicity (2, 3). S1PRs are highly expressed on cardiomyocytes; the initial agonistic effect of FTY720-P on S1PRs before inducing their internalization seems to be responsible for its dose-dependent cardiotoxic effects (5). Hepatotoxic dose-dependent effects have also been reported (2, 3, 6), deemed to be associated with FTY720's metabolic burden on the liver (7). Other dose-dependent unwanted and toxic effects of FTY720 due to S1PR agonism, e.g., macular edema (8), unwanted loss of weight and appetite (9), etc. have been reported.

FTY720 itself was derived from myriocin (ISP-1) – a secondary metabolite of the entomopathogenic fungus *Isaria sinclairii* and a potent inhibitor of the serine palmitoyltransferase (SPT) enzyme in the *de-novo* sphingolipid biosynthesis pathway (10). Myriocin alters a metabolic pathway in eukaryotic cells vital for their stability and proliferation. Consistently, it has shown to have antifungal effects e.g., against *Candida* and *Aspergillus spp.* (11, 12).

While FTY720 has no activity against SPT, emerging studies (13, 14) are pointing towards S1PR-independent anti-proliferative effects of FTY720 in its non-phosphorylated form. Meanwhile, several cases of opportunistic fungal infections are reported among people receiving FTY720 chronically (15, 16). Although these reports describe case studies and population-based evidence is lacking in this regard, it seems unclear whether FTY720 facilitates fungal expansion *in vivo* by weakening the immune system, or has retained the antifungal effect of its molecular ancestor.

The subject of identifying and confirming the possible antifungal properties of substances like FTY720 – which could be administered safely ~~in~~ in ~~homo~~ humans – gains more relevance when considering the rapid emergence of serious mycosis cases resistant to our usual antifungal armamentarium. Among the candidiasis cases, resistance to drugs is classically attributed to the less prevalent infections with non-

Commented [MOU4]: Reviewer 1 comment 5 toxicity related studies were incorporated, cited, and discussed in the manuscript per reviewer's request.

Formatted: Font: Not Italic

Formatted: Font: Not Italic

Formatted: Font: Not Italic

albicans species, however, recent studies are documenting an alarming increase in the antifungal resistance of *Candida (C.) albicans* – the dominant pathogen in candidiasis cases (17, 18). As the incidence of opportunistic candidiasis is rising, and commensal *C. albicans* has a high prevalence (19), its resistance to drugs could be a major complicating factor of the future candidiasis cases.

Therefore, we first aimed to determine whether FTY720 has antifungal effects *in vitro* using antifungal susceptibility testing. Then, we aimed to characterize its effects on immunological responses and fungal burden values in mice infected with the opportunistic yeast *C. albicans* – one of the most prevalent causative agents of systemic opportunistic mycosis. We hereby report the study in accordance with the Animal Research: Reporting of In Vivo Experiments 2.0 (ARRIVE 2.0) guidelines (available at: https://arriveguidelines.org/).

2. Methods

2.1. Design

The present study was conducted from October 2019 until July 2020 in the Islamic Azad University of Falavarjan, Isfahan, Iran. It could be divided into an *in-vitro* substudy and an *in-vivo* substudy, with the former aiming to measure FTY720's minimum inhibitory concentration (MIC) for *C. albicans*, and the later aiming to characterize its effect on the immune response measures and fungal burden values in mouse candidiasis models. The later substudy (from now on referred to as the *in-vivo* study) was designed with the following formulation of research question:

- Population: female C57BL/6 mice.
- Interventions:
  - Oral FTY720
  - Intraperitoneal (IP) *C. albicans* inoculum
- Comparison:
  - Oral normal saline (oral placebo)
  - IP normal saline (injectable placebo)
- Outcomes and rationale of each:
  - Complete blood count with differential (CBC diff) and serum concentrations of interferon-gamma (IFNγ) and interleukin-10 (IL10) after 21 days of receiving oral FTY720/placebo: to characterize the immune responses of the mice.
  - Colony count of *C. albicans* in samples from the vaginas, kidneys, and livers of the mice after 21 days of receiving oral FTY720/placebo: to measure the fungal burden (20).

Based on the formulation of the research question, the *in-vivo* study was conducted with six arms (Fig.1):

Formatted: Font color: Text 1

1. Control: Six mice receiving oral and injectable placebo.
2. Only FTY720: Six mice receiving oral FTY720 and injectable placebo.

3. Only *C. albicans*: Six mice receiving oral placebo and IP *C. albicans* inoculum.
4. FTY720 after *C. albicans*: Ten mice receiving oral FTY720 from two days after IP *C. albicans* inoculum.
5. FTY720 with *C. albicans*: Ten mice receiving oral FTY720 and IP *C. albicans* inoculum on the same day.
6. FTY720 before *C. albicans*: Ten mice receiving oral FTY720 from two days before receiving IP *C. albicans* inoculum.

The only exclusion criterion for the *in-vivo* study was death due to an unrelated reason. No other eligibility criteria were considered.

[Figure 1 placeholder]

2.2. In-vitro antifungal susceptibility testing

2.2.1. Well diffusion assay

We first used a *qualitative* well diffusion assay in order to i) estimate the probable FTY720's *in-vitro* MIC for *C. albicans* and ii) to have an estimate of the drug serial concentration measures required for the following microbroth dilution assay. For the well diffusion assay, a 0.5 McFarland standard inoculum of *C. albicans* (CBS2747, Tehran University, Iran) was spread on three plates of Sabouraud dextrose agar (SDA) (Scharlau, Turkey). A total of eleven sample wells (four wells in two plates, and three wells in one plate) each with a diameter of six millimeters were created in the plates using a sterile Pasteur pipette. One well was filled with normal saline as control while the others were filled with 200 µL of FTY720 suspensions with serial concentrations from 0.005 to 1.5 mg/mL. The visual presence of a zone of inhibition around each well was determined after a 30-hour incubation at 37 °C. The lowest concentration for which a zone of inhibition surrounded the corresponding well was considered as the probable MIC.

Commented [MOU5]: Reviewer 1 comment 3
The required space between values and units were added.

Commented [MOU6]: Reviewer 1 comment 3
The required space between values and units were added.

2.2.2. Microbroth dilution assay

After obtaining the probable MIC using the well diffusion assay, for precise and standardized measurement the MIC, a microbroth dilution assay – the gold-standard method for MIC measurement according to almost all standard laboratory guidelines – was performed following the methods described by the The European Committee on Antimicrobial Susceptibility Testing (EUCAST) (21). Briefly, microtubes in a sterile microtube plate were filled with 10 µL of a 0.5 McFarland standard inoculum of *C. albicans*, 90 µL of Sabouraud dextrose broth (SDB) (Scharlau, Turkey), and 100 µL of serially-diluted suspensions of FTY720, from 0.0005 to 0.5 mg/mL (i.e., the microtubes finally contained FTY720 suspensions with serial dilutions from 0.00025 to 0.25 mg/mL). One microtube was added by normal saline instead of FTY720 (positive control), and one was filled with sterile SDB without the *C. albicans* inoculum (negative control). The optical density (OD) of each microtube was read and documented at 640 nm immediately after preparation, and after a

Commented [MOU7]: Reviewer 1 comment 3
The required space between values and units were added.

Commented [MOU8]: Reviewer 1 comment 3
The required space between values and units were added.

Commented [MOU9]: Reviewer 1 comment 3
The required space between values and units were added.

Commented [MOU10]: Reviewer 1 comment 3
The required space between values and units were added.

24-hour incubation at 37 °C. The minimum drug concentrations for which the post-incubation change in OD was <1% and <50% were considered as the MIC_{99} and the MIC_{50}, respectively.

2.3. Preparation, allocation, and follow-up of the mice

For the *in-vivo* study, 48 female C57BL/6 mice (Royan Institute, Iran) – each weighing 22 (±3) grams – were prepared in accordance with the National Research Council guidelines for laboratory animal care (22). All mice were kept for one week before randomization, in standard cages in a humidity-controlled room at a 25 °C (±2) temperature and a 12 hours daylight, 12 hours darkness cycle. Thereafter, each mouse was identification-marked by an ear tag, and allocated to each one of the study arms based on a random sequence generated by the NumPy software package (for Python version 2.7 on MacOS). They received their interventions in an open-label manner and were followed up daily for 21 days after starting oral FTY720/placebo. FTY720 (0.3 mg/kg/day) or oral placebo was administered by gastric gavage in form of a 100 μL suspension. For determination of frequency and dosage of FTY720, we selected a regimen frequently used in previous studies, proven to bear immunomodulatory effects, and associated with minimal adverse reactions in C57BL/6 mice (23-29). The mice assigned to be infected with *C. albicans* were intraperitoneally injected with an inoculum containing 1.5 x 10^5 colony forming units (CFU) of *C. albicans*, while the others were injected with normal saline in the same amounts. At the end of the follow-up, the mice were sedated with ketamine-xylazine (100-10 mg/kg) and 1 mL of blood was taken from each through cardiac catheterization. The blood samples were taken in anticoagulated tubes, and were promptly sent to be prepared for CBC diff and cytokine assays. Then, through sterile procedures, the mice's livers, kidneys, and vaginal samples were extracted and sent to be prepared for fungal culture. The mice were, thereafter, euthanized with anesthetic overdose per American Veterinary Medical Association (AVMA) guidelines (30).

2.4. Analyses of samples

In order to characterize the mice's immune responses, absolute WBC and lymphocyte counts, serum IL10 (an anti-inflammatory cytokine), and IFNγ (a proinflammatory cytokine) concentrations were measured. CBC diff was performed on whole blood samples by manual hemocytometry (Neubauer chamber) (31). IFNγ and IL10 concentrations were quantified using ELISA (BioAssay™ kit for mouse, USBiological, USA) in accordance with the manufacturer's instructions. For fungal culture, the vaginal samples were directly spread on SDA plates. The livers and kidneys were first homogenized and filtered (using sterile techniques and equipment), were diluted in ten serial concentrations, and then spread on SDA plates. After a 30-hour incubation at 37 °C, the number of CFU's were counted and documented.

2.5. Statistical methods

2.5.1. Data preparation and determination of appropriate tests

The distributions of immune response measures (IFNγ, IL10. WBC, and ALC) were assumed to be lognormal; these measures were log_{10}-transformed for the analyses. The distribution of fungal burden

Commented [MOU11]: Reviewer 1 comment 3
The required space between values and units were added.

Commented [MOU12]: Reviewer 1 comment 3
The required space between values and units were added.

Commented [MOU13]: Reviewer 1 comment 3
The required space between values and units were added.

Commented [MOU14]: Reviewer 1 comment 3
The required space between values and units were added.

Commented [MOU15]: Reviewer 1 comment 3
The required space between values and units were added.

Commented [MOU16]: Reviewer 1 comment 3
The required space between values and units were added.

values was assumed to be normal. The normality/lognormality assumptions were tested using the Kolmogorov-Smirnov method (32, 33); if the assumptions were confirmed by alpha>0.05, parametric tests (e.g., one-way analysis of variance [ANOVA]), and if not, non-parametric tests (e.g., Kruskal-Wallis) were used for those measures. Post-hoc and correction for multiple comparisons was performed by controlling the false discovery rate, using the two-stage step-up method of Benjamini, Krieger, and Yakutielli (34).

In order to determine whether a single pooled variance could be used in the parametric comparisons, the group variances were compared using the Brown-Forsyth test (35). In cases of insignificant (P>0.05) difference among variances of the arms, a single pooled variance was calculated and used; otherwise, the Welch and Brown-Forsyth method (36) was used (variances were calculated for individual arms separately).

2.5.2. Analyses and their rationale

To confirm if the follow-up period was sufficient for FTY720 to implement its immunological effects, the IFNγ, IL10, WBC, and ALC measures were compared between the control arm (arm 1) and the arm receiving FTY720 with injectable placebo (arm 2).

To confirm if the follow-up period was sufficient for the mice to develop an immune response against the fungal infection, the IFNγ, IL10, WBC, and ALC measures in the control arm (arm 1) were compared with the arm receiving IP *C. albicans* inoculum with oral placebo (arm 3).

To confirm if the follow-up period was sufficient for *C. albicans* to disseminate *in vivo*, the fungal burden values in the control arm (arm 1) were compared with the arm receiving IP *C. albicans* inoculum with oral placebo (arm 3).

To characterize the effect of FTY720 on mice's immunological response to *C. albicans*, the IFNγ, IL10, WBC, and ALC measures in the arms 4,5,6 (i.e., the arms receiving both FTY720 and IP *C. albicans*) were once compared with the arm receiving FTY720 with injectable placebo (arm 2), and once with the arm receiving IP *C. albicans* with oral placebo (arm 3).

In order to characterize the effect of FTY720 on the fungal burden *in vivo,* the liver, kidney, and vaginal colony count values in arms 4,5,6 (i.e., the arms receiving both FTY720 and IP *C. albicans)* were compared with the arm receiving IP *C. albicans* with oral placebo (arm 3).

2.5.3. Software

The Prism software (version 9.0.2 for MacOS; GraphPad Software LLC.) was used for statistical analysis and graphing.

2.6. Reproducibility, safety and e~~E~~thical considerations

In order to ensure the reproducibility of the results, the Enhancing the QUAlity and Transparency Of health Research (EQUATOR) guidelines (37) were followed to report the study, enabling all researchers across the globe to repeat the experiments, obtain, compare, and validate the results.

Commented [MOU17]: Reviewer 2 comment 1
Our effort to enable adequate reproducibility was highlighted.

The laboratory researchers followed strict regulatory standards in terms of safety; microbiological Petri dishes were not handled without gloves during any of the experiments, and the laboratory researchers wore sterile gowns, protective face-shields and masks, as well as following standard hand-washing protocols with povidone iodine solution before entering and after exiting the laboratory. All of the safety procedures were approved by the regulatory scientific review board who were monitoring the experiments through their surveillance systems.

The animal subjects of this study were kept in standard cages and were not restricted in terms of space, water, and food. All procedures on animal subjects were executed in accordance with national ethical guidelines. This study was approved by the ethics committee of the Islamic Azad University Falavarjan branch (Approval ID: IR.IAU.FALA.REC.1401.010).

3. Results

3.1. In-vitro antifungal susceptibility tests

In the well diffusion assay, the lowest concentration of FTY720 for which a visible zone of inhibition surrounded its corresponding well was 0.5 mg/mL (~~e~~Fig.2~~1~~), hence, the probable MIC of FTY720 was estimated to be 0.5 mg/mL or lower. As mentioned, we then used the resulted qualitative measures for precise and standard MIC measurement with a microbroth dilution assay, in which the MIC_{99} was measured to be 0.25 mg/mL, and the MIC_{50} was measured to be 0.12 mg/mL (Fig.3~~2~~). These results confirmed that FTY720 comprises antifungal effects *in vitro*.

[Figure 2 placeholder]

[Figure 3~~.2~~ placeholder]

3.2. Overview and validation of the in-vivo study

The study was conducted per protocol; no unintended events happened during the study. The mice were randomized and allocated to the study arms two days before receiving their oral interventions (T -2). All of the randomized mice completed were followed up for 23 days (until T +21), except for one mouse in the control arm (arm 1), which died due to an unknown reason in the second day of oral normal saline consumption (T +2, four days after randomization). An investigative dissection of the mouse was conducted to identify the possible reason of death, but no gross abnormalities were detected. Samples from the normal saline administered to the mouse was cultured, resulting in growth of no microorganisms after one week. Apart from the signs of sepsis in the mice receiving IP *C. albicans* inoculum, the other mice experienced no serious adverse events during their follow-up.

Commented [MOU18]: Reviewer 2 comment 3
More descriptions on the safety considerations were added to address any concern in this regard.

Commented [MOU19]: Reviewer 1 comment 3
The required space between values and units were added.

Formatted: Font color: Text 1

Commented [MOU20]: Reviewer 1 comment 3
The required space between values and units were added.

Commented [MOU21]: Reviewer 1 comment 3
The required space between values and units were added.

Commented [MOU22]: Reviewer 1 comment 3
The required space between values and units were added.

Formatted: Font color: Text 1

Formatted: Font color: Text 1

At the end of the study, the immunological profile of the mice treated with FTY720 and injectable placebo (arm 2) significantly differed from the controls (arm 1) (Fig.43a), indicating a sufficient follow-up period for FTY720 to execute its immunomodulatory effects. FTY720 significantly decreased IFNγ, increased IL10, and decreased white blood cell (WBC) and absolute lymphocyte (AL) counts (Fig.43a).

The *C. albicans*-infected mice receiving oral placebo (arm 3) showed significantly different immunological profiles and fungal burden measures with the controls (arm 1) (Fig.43a); this indicated that the follow-up period was sufficient for dissemination of *C. albicans in vivo,* and for an antifungal immunological response to develop. Disseminated candidiasis significantly increased IFNγ, WBC, and AL counts but insignificantly affected IL10 (Fig.43a). Cultures of liver, kidney, and vaginal samples of the mice not receiving IP *C. albicans* showed no growth of fungi, whereas the samples from all of the *C. albicans*-injected ones showed growth of *C. albicans* – confirmed with gross visual examination and direct smear microscopy (eFig.52, eFig.63). In two cultures (one kidney sample from a mouse in arm 3 and one vaginal sample from a mouse in arm 6) multiple microorganisms were colonized, highly indicative of procedural contamination; they were excluded from analyses.

[Figure .43 placeholder]

[Figure 5 placeholder]

[Figure 6 placeholder]

3.3. Effect of FTY720 on mice's immune response to C. albicans

In order to characterize the effect of FTY720 on the mice's immune response to *C. albicans*, we compared the arms receiving the IP *C. albicans* inoculum before, after, or simultaneously with initiation of FTY720 (arms 4,5,6), with the ones receiving FTY720/injectable placebo or *C. albicans*/oral placebo (arms 2,3) (Fig.1).

Similar to the mice receiving IP *C. albicans*/oral placebo (arm 3), *C. albicans* inoculation before, and simultaneously with initiation of FTY720 (arms 4,5) significantly increased IFNγ levels compared to the mice receiving FTY720/IP placebo (arm 2) (Fig.43b) (eTable 1). However, *C. albicans* inoculation after two days of FTY720 consumption (in arm 6) caused an insignificant increase in IFNγ levels; IFNγ levels were significantly lower in these mice compared to the mice receiving *C. albicans*/oral placebo (arm 3) (Fig.43b) (eTable 1). In simple words, being on FTY720 was required for at least two days at the time of candidiasis induction, for it to hinder the IFNγ response of the mice to the disseminating fungal infection.

The mice infected before initiation of FTY720 (arm 4) had significantly lower levels of IL10 compared to the ones receiving FTY720/IP placebo (arm 2), while being comparable to the ones receiving IP *C. albicans*/oral placebo (arm3) (Fig.3b4b) (eTable 1). It could be interpreted that the prior fungal infection hindered the IL10-increasing effect of FTY720. Induction of candidiasis simultaneously with, or later than FTY720 initiation (in arms 5,6) did not affect the IL10-increasing effect of FTY720 as shown in Figure 43b.

Formatted: Font color: Text 1

Formatted: Font color: Text 1

Formatted: Font color: Text 1

Formatted: Font color: Text 1

Formatted: Font color: Text 1

Formatted: Font color: Text 1

Formatted: Font color: Text 1

Commented [MOU23]: Reviewer 1 comment 2
All eFigures were incorporated into the main text

Formatted: Font color: Text 1

Formatted: Font color: Text 1

Formatted: Font color: Text 1

Formatted: Font color: Text 1

Formatted: Font color: Text 1

As interpreted before, FTY720 significantly decreased, and candidiasis significantly increased WBC and AL counts in mice (Fig.43a). The mice which started taking FTY720 before or after candidiasis induction (arms 4,6) showed significantly lower WBC and AL counts than the ones receiving IP *C. albicans*/oral placebo (arm 2) (Fig.43b). Surprisingly, the mice with simultaneous initiation of FTY720 and induction of candidiasis still showed significantly increased WBC and AL counts – significantly higher than the mice receiving FTY720/injectable placebo (arm 2), and comparable to the ones receiving *C. albicans*/oral placebo (arm 3) (Fig.43b) (eTable 1).

3.4. Effect of FTY720 on fungal burden *in vivo*

To demonstrate the antifungal effect of FTY720 *in vivo*, we cultured specimens of mice's livers, kidneys, and vaginas 21 days after initiation of FTY720/oral placebo, and compared the resulted fungal burden values between the mice receiving *C. albicans*/oral placebo (arm 3), and the ones receiving both FTY720 and IP *C. albicans* inoculum (arms 4,5,6). The mice receiving FTY720 before, after, or simultaneously with induction of candidiasis showed significantly lower fungal burden measures in their livers and kidneys (eTable 1) (Fig.74); interestingly, the ones initiating FTY720 and getting infected on the same day (arm 5) – which also showed the high AL counts before – had the lowest liver/kidney fungal burdens – significantly lower than all of the other arms. Regarding the vaginal burdens, only the mice initiating FTY720 before receiving the IP *C. albicans* inoculum (arm 6) showed lower values than the ones receiving IP *C. albicans*/oral placebo (arm 3) (eTable 1) (Fig.74).

[Figure 7 4 placeholder]

4. Discussion

Our results demonstrated that FTY720 has antifungal effects against *C. albicans* – both *in vitro* and *in vivo*. In the mice models of disseminated candidiasis, this effect varied based on the chronological relation of FTY720 initiation and *C. albicans* inoculation. Our report is the first describing the *in-vivo* antifungal effect of FTY720 as far as we know.

In line with our study, a recent *ex-vivo* study by Wei and colleagues pointed to the fungicidal effect of FTY720, which synergized the effect of amphotericin B (38). Wei et al. showed that this fungicidal effect is present against non-albicans Candida spp., *Saccharomyces cerevisiae*, and *Cryptococcus neoformans* as well as *C. albicans* (38). They also showed that 0.011 mg/mL of FTY720 affects *C. albicans'* growth rate similar to 0.08 mg/L of amphotericin B (38). In an attempt to characterize the cause of this effect, they demonstrated that FTY720 induces production and hyperaccumulation of reactive oxygen species (ROS) in a dose-dependent manner; scavenging of the ROS from the samples using N-acetyl cysteine (NAC) compromised the fungicidal effect of FTY720 significantly. Therefore, Wei et al.'s study suggested hyperaccumulation of ROS as the reason of FTY720's fungicidal effect (38). ROS accumulation in fungi

Formatted: Font color: Text 1

Formatted: Font color: Text 1

Formatted: Font color: Text 1

Formatted: Font color: Text 1

Formatted: Font color: Text 1

Formatted: Font color: Text 1

Commented [MOU24]: Reviewer 1 comment 3
The required space between values and units were added.

Commented [MOU25]: Reviewer 1 comment 3
The required space between values and units were added.

induced by unphosphorylated FTY720 has been observed in previous studies as well (14, 39), the mechanism of which is an area of active investigation.

Administration of FTY720 at 1.25 mg/day in healthy adults – above which has proved unbeneficial for people with MS (6) – results in a maximum blood concentration (C_{max}) of 10.2 (±2.7) ng/mL at steady-state (7); this concentration is 25000 times lower than its MIC_{99} for *C. albicans.* Maximum concentrations of FTY720 in kidney and liver tissues reach approximately 40 times the C_{max} (40) – still much less than its *in-vitro* MIC_{99}. Therefore, the current FTY720 regimens in people with MS are doubted to directly restrict *C. albicans* infection. In our *in-vivo* study, the mice received 0.3 mg/kg/day of FTY720. A single 0.3 mg/kg dose of FTY720 is associated with a maximum liver and kidney concentration of around 1.5 µg/mL (40). Considering a 10-fold steady-state concentration reached by daily administration, the maximum concentration of FTY720 in liver and kidney reaches 15 µg/mL – 17 times less than its *in-vitro* MIC_{99} and 8 times less than its MIC_{50}. Despite this fact, we did observe the antifungal effect of FTY720 *in vivo*. Hence, we speculate that FTY720 had a synergistic effect with host's innate immunity against *C. albicans*. The yeast could antagonize oxidative stress up to a specific threshold by inducing antioxidant metabolic pathways (41). The innate immunity is absent *in vitro*, therefore, higher concentrations of FTY720 are needed to raise the ROS levels up to the threshold intolerable by the yeast. *In vivo*, the generated ROS induced by FTY720, although may not be adequate by itself, is added by the ROS produced by the innate immune cells, while also attracting more cells to the frontline (42), facilitating their phagocytizing, and easing the killing of fungi by them (38). Nevertheless, countless mechanistic factors are involved in fungal proliferation and expansion *in vivo*; determination of our observations' mechanistic background could be an interesting subject for future studies.

As mentioned in the introduction, some case studies focusing on atypical cryptococcal infections (15, 16) hypothesize that people with MS receiving FTY720 may be generally more susceptible to fungal infections. However, Wei et al. demonstrated that the *ex-vivo* fungicidal effect of FTY720 against *Cryptococcus neoformans* is similar to its effect on *C. albicans,* and we demonstrated that the *in-vitro* antifungal effect of FTY720 against *C. albicans* is also present *in vivo.* The dose-dependency of FTY720's antifungal effect may be the explanation behind this discrepancy. It could be hypothesized that FTY720's immunosuppressive effect when administered chronically with low doses – as in people with MS – facilitates fungal infections, as its antifungal effects are absent in concentrations reached with those regimens (discussed above).

Another notable observation in our study was the mice with simultaneous C. albicans inoculation and initiation of FTY720 (arm 5) had a prominent increase in AL counts, similar to the mice receiving *C. albicans*/oral placebo (arm 3); however, their kidney and liver samples revealed significantly lower fungal burdens. The decreased fungal burden values in these mice could not merely be explained by the increased AL counts, otherwise, the mice receiving *C. albicans*/oral placebo (arm 3) who had similar AL counts would

Commented [MOU26]: Reviewer 1 comment 3
The required space between values and units were added.

Commented [MOU27]: Reviewer 1 comment 3
The required space between values and units were added.

Commented [MOU28]: Reviewer 1 comment 3
The required space between values and units were added.

Commented [MOU29]: Reviewer 1 comment 3
The required space between values and units were added.

Commented [MOU30]: Reviewer 1 comment 3
The required space between values and units were added.

have similarly shown low fungal burdens in their organs. Further fundamental work is warranted to explain this interesting observation.

5. Conclusion

We showed that the immunomodulatory drug FTY720 has an antifungal effect *in vitro*, which is present at even lower concentrations *in vivo*. The facilitation of ROS hyperaccumulation by FTY720 – which assists the innate immune system in creating an antifungal environment – may explain our observations. Future studies are warranted to evaluate further aspects of repositioning FTY720 for antifungal use in clinical settings.

6. Limitations

In order to ensure a sterile and prompt procedure of homogenization and fungal culturing, we regret that we did not perform any sectioning, staining, and direct microscopy of the liver, kidney, and vagina samples to provide the visual confirmation of *C. albicans* colonization *within* the tissues of the mice in treated and control groups. Also, mainly due to limited resources, the *in-vitro* effect of FTY720 on important virulence traits of *C. albicans,* including filamentation, adhesion, and biofilm formation was not investigated in any standardized manner. Furthermore, the photographic documentations of the experiments provided in this report may not be considered in accordance with research standards; therefore, providing research-grade photographic documentations of the experiments conducted in our study and their visual validation future studies is warranted to contribute to the reproducibility of the results. Other limitations included outcome measurement at a single timepoint rather than several timepoints during the study, usage of limited number of assays, relatively short follow-up period, open-label fashion, not measuring drug concentrations in blood and tissues after administration, not measuring the ROS concentrations *in vitro* or *in vivo*, and other possible unnoticed biases. Further replicative studies are encouraged to add to our knowledge and account for the limitations of our study.

7. Conflict of interest and funding

None.

8. Author contributions

NN: conceptualization; methodology; investigation; data curation; resources; writing – original draft; validation; project administration.

MM: supervision; validation.

ME: supervision; writing – review and editing.

NS: methodology; formal analysis; visualization; writing – original draft; writing – review and editing.

9. Acknowledgements

None.

10. Data availability

All data produced in this study are presented within the manuscript, its figures, and supplemental contents. Raw data is also available upon reasonable request from the corresponding author.

11. References

1. Roy R, Alotaibi AA, Freedman MS. Sphingosine 1-Phosphate Receptor Modulators for Multiple Sclerosis. CNS Drugs. 2021;35(4):385-402.
2. Kappos L, Radue E-W, O'Connor P, Polman C, Hohlfeld R, Calabresi P, et al. A placebo-controlled trial of oral fingolimod in relapsing multiple sclerosis. New England Journal of Medicine. 2010;362(5):387-401.
3. La Mantia L, Tramacere I, Firwana B, Pacchetti I, Palumbo R, Filippini G. Fingolimod for relapsing-remitting multiple sclerosis. Cochrane Database Syst Rev. 2016;4:Cd009371.
4. Wu X, Xue T, Wang Z, Chen Z, Zhang X, Zhang W, et al. Different Doses of Fingolimod in Relapsing-Remitting Multiple Sclerosis: A Systematic Review and Meta-Analysis of Randomized Controlled Trials. Front Pharmacol. 2021;12:621856.
5. Vargas WS, Perumal JS. Fingolimod and cardiac risk: latest findings and clinical implications. Ther Adv Drug Saf. 2013;4(3):119-24.
6. Wu X, Xue T, Wang Z, Chen Z, Zhang X, Zhang W, et al. Different doses of fingolimod in relapsing-remitting multiple sclerosis: a systematic review and meta-analysis of randomized controlled trials. Frontiers in pharmacology. 2021;12:1132.
7. David OJ, Kovarik JM, Schmouder RL. Clinical pharmacokinetics of fingolimod. Clinical pharmacokinetics. 2012;51(1):15-28.
8. Cugati S, Chen CS, Lake S, Lee AW. Fingolimod and macular edema: Pathophysiology, diagnosis, and management. Neurol Clin Pract. 2014;4(5):402-9.
9. Etemadifar M, Khorvash R, Zolfagharifard H, Sedaghat N, Nouri H, Salari M. Weight Loss and Loss of Appetite as a Side Effect of Fingolimod. 2022.
10. Chiba K, Adachi K. Discovery of fingolimod, the sphingosine 1-phosphate receptor modulator and its application for the therapy of multiple sclerosis. Future Med Chem. 2012;4(6):771-81.
11. Perdoni F, Signorelli P, Cirasola D, Caretti A, Galimberti V, Biggiogera M, et al. Antifungal activity of Myriocin on clinically relevant Aspergillus fumigatus strains producing biofilm. Bmc Microbiology. 2015;15(1):1-8.
12. Yang X, Pei Z, Hu R, Zhang Z, Lou Z, Sun X. Study on the Inhibitory Activity and Possible Mechanism of Myriocin on Clinically Relevant Drug-Resistant Candida albicans and Its Biofilms. Biol Pharm Bull. 2021;44(3):305-15.
13. Zhang L, Wang H-D, Ji X-J, Cong Z-X, Zhu J-H, Zhou Y. FTY720 for cancer therapy. Oncology reports. 2013;30(6):2571-8.
14. Takasaki T, Hagihara K, Satoh R, Sugiura R. More than just an immunosuppressant: the emerging role of FTY720 as a novel inducer of ROS and apoptosis. Oxidative medicine and cellular longevity. 2018;2018.
15. Carpenter AF, Goodwin SJ, Bornstein PF, Larson AJ, Markus CK. Cutaneous cryptococcosis in a patient taking fingolimod for multiple sclerosis: here come the opportunistic infections? Multiple Sclerosis Journal. 2017;23(2):297-9.

16. Del Poeta M, Ward BJ, Greenberg B, Hemmer B, Cree BA, Komatireddy S, et al. Cryptococcal Meningitis Reported With Fingolimod Treatment: Case Series. Neurology-Neuroimmunology Neuroinflammation. 2022;9(3).
17. Whaley SG, Berkow EL, Rybak JM, Nishimoto AT, Barker KS, Rogers PD. Azole antifungal resistance in Candida albicans and emerging non-albicans Candida species. Frontiers in microbiology. 2017;7:2173.
18. Coste AT, Kritikos A, Li J, Khanna N, Goldenberger D, Garzoni C, et al. Emerging echinocandin-resistant Candida albicans and glabrata in Switzerland. Infection. 2020;48(5):761-6.
19. Romo JA, Kumamoto CA. On Commensalism of Candida. J Fungi (Basel). 2020;6(1).
20. Conti HR, Huppler AR, Whibley N, Gaffen SL. Animal models for candidiasis. Curr Protoc Immunol. 2014;105:19.6.1-.6.7.
21. EUCAST DEFINITIVE DOCUMENT E.DEF 7.3.1 Method for the determination of broth dilution minimum inhibitory concentrations of antifungal agents for yeasts. European Committee for Antimicrobial Susceptibility Testing (EUCAST). 2017.
22. Council NR. Guide for the care and use of laboratory animals. 2010.
23. Abdullah CS, Li Z, Wang X, Jin ZQ. Depletion of T lymphocytes ameliorates cardiac fibrosis in streptozotocin-induced diabetic cardiomyopathy. Int Immunopharmacol. 2016;39:251-64.
24. Bonfiglio T, Olivero G, Merega E, Di Prisco S, Padolecchia C, Grilli M, et al. Prophylactic versus Therapeutic Fingolimod: Restoration of Presynaptic Defects in Mice Suffering from Experimental Autoimmune Encephalomyelitis. PLoS One. 2017;12(1):e0170825.
25. Cheng Q, Li D, Liang H, Yang H, Lei D, Gao D, et al. Effects of long-term administration of low-dose FTY720 on survival of murine cardiac allograft. J Huazhong Univ Sci Technolog Med Sci. 2012;32(2):199-204.
26. Efstathopoulos P, Kourgiantaki A, Karali K, Sidiropoulou K, Margioris AN, Gravanis A, et al. Fingolimod induces neurogenesis in adult mouse hippocampus and improves contextual fear memory. Transl Psychiatry. 2015;5(11):e685.
27. Hwang MW, Matsumori A, Furukawa Y, Ono K, Okada M, Iwasaki A, et al. FTY720, a new immunosuppressant, promotes long-term graft survival and inhibits the progression of graft coronary artery disease in a murine model of cardiac transplantation. Circulation. 1999;100(12):1322-9.
28. Pinschewer DD, Ochsenbein AF, Odermatt B, Brinkmann V, Hengartner H, Zinkernagel RM. FTY720 immunosuppression impairs effector T cell peripheral homing without affecting induction, expansion, and memory. The Journal of Immunology. 2000;164(11):5761-70.
29. Wang G, Kim RY, Imhof I, Honbo N, Luk FS, Li K, et al. The immunosuppressant FTY720 prolongs survival in a mouse model of diet-induced coronary atherosclerosis and myocardial infarction. J Cardiovasc Pharmacol. 2014;63(2):132-43.
30. Underwood W, Anthony R. AVMA guidelines for the euthanasia of animals: 2020 edition. Retrieved on March. 2020;2013(30):2020-1.
31. Database. JSE. Basic Methods in Cellular and Molecular Biology. Using a Hemacytometer to Count Cells. JoVE, Cambridge, MA,. 2020.
32. Smirnov N. Table for Estimating the Goodness of Fit of Empirical Distributions. The Annals of Mathematical Statistics. 1948;19(2):279-81, 3.
33. Kolmogorov A. Sulla determinazione empirica di una lgge di distribuzione. Inst Ital Attuari, Giorn. 1933;4:83-91.
34. Benjamini Y, Krieger AM, Yekutieli D. Adaptive linear step-up procedures that control the false discovery rate. Biometrika. 2006;93(3):491-507.
35. Brown MB, Forsythe AB. Robust tests for the equality of variances. Journal of the American statistical association. 1974;69(346):364-7.
36. WELCH BL. THE GENERALIZATION OF ‘STUDENT'S’ PROBLEM WHEN SEVERAL DIFFERENT POPULATION VARLANCES ARE INVOLVED. Biometrika. 1947;34(1-2):28-35.
37. Groves T. Enhancing the quality and transparency of health research. British Medical Journal Publishing Group; 2008.
38. Wei L-Q, Tan J-C, Wang Y, Mei Y-K, Xue J-Y, Tian L, et al. Fingolimod potentiates the antifungal activity of amphotericin B. Frontiers in cellular and infection microbiology. 2021;11:346.
39. Hagihara K, Mizukura A, Kitai Y, Yao M, Ishida K, Kita A, et al. FTY 720 stimulated ROS generation and the S ty1/A tf1 signaling pathway in the fission yeast S chizosaccharomyces pombe. Genes to Cells. 2014;19(4):325-37.

40. Meno-Tetang GM, Li H, Mis S, Pyszczynski N, Heining P, Lowe P, et al. Physiologically based pharmacokinetic modeling of FTY720 (2-amino-2 [2-(-4-octylphenyl) ethyl] propane-1, 3-diol hydrochloride) in rats after oral and intravenous doses. Drug metabolism and disposition. 2006;34(9):1480-7.
41. Garcerá A, Casas C, Herrero E. Expression of Candida albicans glutathione transferases is induced inside phagocytes and upon diverse environmental stresses. FEMS yeast research. 2010;10(4):422-31.
42. Brothers KM, Gratacap RL, Barker SE, Newman ZR, Norum A, Wheeler RT. NADPH oxidase-driven phagocyte recruitment controls Candida albicans filamentous growth and prevents mortality. PLoS pathogens. 2013;9(10):e1003634.

Figure legends

Figure 1: Schematic overview of the *in-vivo* study arms.

Figure 2: Photographs of well diffusion plates after incubation. The wells 10 to 1 contained FTY720 with serial concentrations from 0.005 to 1.5 mg/mL respectively. The control "cont" well contained normal saline. Zones of inhibition are visible surrounding the wells 4 to 1, corresponding to serial concentrations from 0.5 to 1.5 mg/mL.

Figure ~~2~~3: Results of the microbroth dilution assay for determining FTY720's MIC for *C. albicans*. The measured plotted on the Y-axis are resulted from subtracting the pre-incubation (background) from the post-incubation OD's. The hyphenated line corresponds to the best-fit regression model. Abbreviations: OD, optical density.

Figure ~~3~~4: Immune profile of the mice at the end of the *in-vivo* study stratified by study arms. **(a)** comparison of immune response measures between the control mice (arm 1), the mice receiving FTY720/injectable placebo (arm 2), and the ones receiving IP *C. albicans* inoculum/oral placebo (arm 3). These measures confirm that the follow-up duration of the *in-vivo* study was sufficient. **(b)** comparison of immune response measures between the mice receiving FTY720/injectable placebo (arm 2), and the mice receiving IP *C. albicans* inoculum/oral placebo (arm 3), and the treated infected mice (arms 4,5,6); only comparisons with P<0.05 are shown. ns: P>0.05; *P<0.05; **0.05<P<0.01; ***0.01<P<0.001; ****P<0.0001. Abbreviations: IFNγ, interferon-gamma; IL10, interleukin-10; WBC, white blood cell; ALC, absolute lymphocyte count; IP, intraperitoneal; C. alb, *Candida albicans.*

Figure 5: Photographs of four selected culture plates *after* completion of the experiments and documentation of their results. It should be noted that *during* the experiments, none of the microbiological Petri dishes were handled without gloves as seen in these photographs. a) Vaginal sample from a mouse in arm 5; b) Liver sample from a mouse in arm 6; c) Vaginal sample from a mouse in arm 2 (above) and a mouse in arm 3 (below); d) Kidney sample from a mouse in arm 3.

Figure 6: Micrograph of gram-stained smears of colonies in vaginal sample cultures of two mice in arm 5. C. albicans yeasts are seen undergoing filamentation.

Figure ~~7~~4: Colony counts of *C. albicans* in kidneys, livers, and vaginas of the infected arms at the end of the *in-vivo* study. ns: P>0.05; *P<0.05; **0.05<P<0.01; ***0.01<P<0.001; ****P<0.0001. Abbreviations: C. alb, *Candida albicans.*

Formatted: Font color: Text 1

Formatted: Font color: Text 1

Formatted: Font color: Text 1

Formatted: Font color: Text 1

Formatted: Font color: Text 1

Formatted: Font: Not Bold

Formatted: Font: Not Bold, Italic

Formatted: Font: Not Bold

Formatted: Font: Not Bold

Formatted: Font: Italic

Commented [MOU31]: Reviewer 2 comment 3
An explanation was added to address any concerns about handling of petri dishes without gloves in these photographs.

Formatted: Font: Not Bold

Formatted: Font color: Text 1

Response to the reviewer comments

Dear Editor

Thank you for having our manuscript reviewed for the second round. We carefully considered each of the reviewer comments, revised and improved our manuscript accordingly. The changes were tracked in a marked version of the revised manuscript, which is uploaded along the clean version. Below, you can find the reviewer comments, along with our responses below each. Please let us know if any further action is required from us. Thank you in advance for your consideration; we hope to hear from you soon.

Best regards

Nahad Sedaghat on behalf of the authors

Reviewer #1:

1. Scientific notation of writing Candida albicans is not correct in abstract. Please check. It should be uniform throughout the manuscript.

Response: Thank you for noticing. We uniformly changed all the instances in the abstract to "*Candida albicans".*

2. Please incorporate the images of well diffusion assay in the manuscript.

Response: Thank you for your suggestion. All eFigures were moved to the main manuscript.

3. Authors should keep space between values and their units throughout the manuscript and used SI units.

Response: Thank you for mentioning. We added the required space between the values and units. About using SI units, as far as we checked, we did not use non-SI units anywhere in the manuscript. In case we overlooked an instance of using any non-SI unit, we would appreciate if you could point to the line, so we could correct that instance.

4. Authors are advised to incorporate images to get better picture of their results.

Response: Thank you for your suggestion. As mentioned, we moved all the eFigures to the main manuscript. We unfortunately have a limitation in presenting further images apart from what is already presented, as the experiments are no longer in progress. We mentioned this point in the limitations section and advised future studies to account for this limitation.

5. Please incorporate toxicity related studies in the manuscript. If already published by others cite that reference in the manuscript.

Response: Thank you for your attention. We added and discussed toxicity related studies as well in the revised manuscript. We hope the changes and improvements satisfy your concerns.

Reviewer #2:

0. The authors have answered have all the questions raised by me.

Response: We are pleased to hear that. Thank you for dedicating your time to our manuscript.

1. However, as the authors mentioned in the article that there are some significant limitations to the reproducibility of the experiments.

Response: Thank you for mentioning your concern about the replicability of the study and reproducibility of the results. In case your concern is due to the lack of standard photographic documentation of the experiments and results, we made the following efforts to account for this limitation and make our report of merit for publication. First of all, we described and disclosed all of the experiments in full detail and in complete accordance with the EQUATOR guidelines, in order to allow their complete repetition by any scientist across the globe. Therefore, replication of the experiments and reproduction of the results would be possible for any team concerned about the lack of proper photographic documentations in the present report. Additionally, all of the experiments were performed in compliance with strict regulations and were being monitored through surveillance systems by independent scientific, safety, and ethical review boards, consisting of anonymous nationally-certified scientists. Finally, we shared and deposited our data for all researchers across the globe to review and validate. We would also welcome any further suggestions to improve our work.

2. The supplementary figures are also not as per scientific standards.

Response: Thank you for mentioning your concern. We completely agree that the presented figures are not in compliance with scientific standards, as we unfortunately, did not intend to keep scientific photographic documentations at the beginning. The reason was that in presence of strict regulations and standard guidelines, we erroneously considered photographic documentations to be optional. Now, as the experiments are no longer in process, we are unfortunately limited in terms of presenting standard figures. However, describing the experiments with full detail allows their repetition by any researcher concerned about the reproducibility of the results. In the limitations section, we explicitly stated this point, and strongly encouraged further replication of the experiments using the detailed description of the methods we provided. We further stated that due to our limitation in providing standard figures, our conclusions are subject to reproduction of the results by future studies with standard photographic documentations.

3. especially handling microbiological Petri dishes without gloves is a serious concern.

Response: Thank you for your punctiliousness. We completely acknowledge your concern. We should mention that the photograph you are referring to was taken *after completion of the experiments and documentation of their results*. *During* the experiments, the laboratory researchers followed strict regulatory standards in terms of their safety; microbiological Petri dishes were not handled without gloves during any of the experiments, and the laboratory researchers wore sterile gowns, protective face-shields and masks,

as well as following standard hand-washing protocols with povidone iodine solution before entering the laboratory. This has been evidenced by absence of biological and non-biological contamination in all of the Petri dishes except for one, and by the approval of the safety procedures by the regulatory scientific review board. These points were clarified in the revised manuscript and in the legend of the mentioned figure to appreciate any concern.

4. There are still a few typos in the manuscript (eg. Line 63 homo-, IFNg instead of γ in the etable etc.).

Response: We apologize for overseeing the mentioned typos in our reviews. The Latin phrase “*in homo*” means “in man” in English; we exchanged “*in homo*” with the standard English term “in humans” to avoid any confusion. IFNg was changed to IFNγ in the supplementary table as well. Furthermore, we reviewed the revised manuscript several more times to ensure proper usage of language for the readers. We would certainly appreciate any further suggestions for improving our report.

5. Therefore, I recommend to resubmit the article with proper figures.

Response: Thank you for your recommendation. As mentioned previously, the experiments are no longer in progress, therefore, we are unfortunately limited in presenting standard photographic documentations. We truly hope our explicit and detailed disclosure of the experimental procedures according to the EQUATOR guidelines and encouragement of future replicative studies could at least, partially resolve your concerns about the reproducibility of the results. We are also, hoping that the publication of our study, if considered of merit based on novelty and relevance, leads to conduction of further studies on the subject accounting for our limitations (including provision of standard figures) and validating the results. Once again, we would like to appreciate your punctiliousness and explicitly in critically appraising our study, and thank you for your time and consideration.
