# Supplementary material for: Immunomodulatory drug fingolimod (FTY720) restricts the growth of opportunistic yeast Candida albicans in vitro and in a mouse candidiasis model
Source: PLoS One. 2022 Dec 7;17(12):e0278488. doi: 10.1371/journal.pone.0278488 (PMC9728862; doi:10.1371/journal.pone.0278488)
Supplement: Attachment — Submitted filename: Res2Rev.docx [file pone.0278488.s003.docx]

Response to the reviewer comments

Dear Editor

Thank you for having our manuscript considered and reviewed. We carefully considered each of the reviewer comments, revised and improved our manuscript accordingly. The changes were tracked in a marked version of the revised manuscript, which is uploaded along the clean version. Below, you can find the reviewer comments, along with our responses below each. Please let us know if any further action is required from us. Thank you in advance for your consideration; we hope to hear from you soon.

Best regards

Nahad Sedaghat on behalf of the authors

## Reviewer #1

1. *“The authors are advised to go through the entire manuscript and correct it for grammatical errors.”*

**Response:** Thank you for reviewing our manuscript and for your constructive suggestions. Per your request, the manuscript was reviewed and edited by a native English scientific writer to ensure proper understanding of the readers.

2. *“Authors should mention protocol number approved by Institutional Ethics Committee (IEC) in the manuscript.”*

**Response:** Thank you for noticing. The approval number issued by the ethics committee was added under the “Ethical considerations” section.

3. *“In in-vitro well diffusion assay, how did the authors manage to load 200ml of sample in the wells. Also, please mention the zone of inhibition values obtained.”*

**Response:** We loaded 200 “μL” of sample to the wells. Thank you for noticing this error, which is now corrected. Furthermore, it should be mentioned that the well diffusion assay was performed qualitatively only to give us an estimate of the MIC and the required concentrations for the more sensitive and objective broth microdilution assay. In other words, only the qualitative presence of the zones was important to us, as we did not aim to report the MICs based on zone of inhibition cut-offs in well diffusion assays. For the same reason, you could interpret that we did not follow the standard 24-hour incubation protocol to enable MIC estimation based on zone cut-offs. We mistakenly mentioned that the diameters of the zones were measured for MIC determination; this point was corrected. The section on fungal susceptibility assays was reworded while explicitly clarifying and emphasizing the mentioned points to avoid any confusion for the readers.

4. *“Please mention the histopathological images of livers, kidneys and vagina obtained after 21 days of treatment to provide visual analysis of Candida colonization between the treated and control groups.”*

**Response:** In order to ensure a sterile and prompt procedure of homogenization and fungal culturing, we regret that we did not perform any histopathological sectioning of the tissues to provide the requested visual analysis of Candida colonization between the treated and control groups. This point was mentioned in the limitations section. To validate the colonization of *C. albicans*, we did perform staining and direct microscopy of the colonies after culturing; the available gross and microscopic photographs were added to the supplemental contents.

5. *“Authors have mentioned that Fingolimod restricts the growth of opportunistic yeast Candida albicans in vitro and in a mouse candidiasis model. Have the authors investigated the in vitro effect of this compound on important virulence traits mainly- hydrolytic enzymes, yeast to hyphae transition, adhesion and biofilm formation. If yes, please mention this in manuscript.”*

**Response:** Unfortunately, mainly due to limited resources, the *in-vitro* effect of FTY720 on important virulence traits of *Candida albicans* was not investigated in any standardized manner. This limitation is now explicitly stated in the limitations section. Thank you once more for dedicating your time to review and improve our manuscript.

## Reviewer #2

0. *“Overall the necessary experiments were performed in this study. However some structural changes are needed.”*

**Response:** Thank you for reviewing our manuscript and providing us with your valuable comments. We hope that we addressed each of your comments sufficiently.

1. *“the in vitro results should precede the in vivo. The figures should follow the same logic.”*

**Response:** Thank you for your suggestion. The figures followed the order they were mentioned in the text in line with the journal requirements. Per your suggestion, we changed the order of the sections 3.1 and 3.2. In the revised manuscript, you could find the in-vitro results in section 3.1, prior to the first description of the in-vivo results in section 3.2. Their corresponding figures also follow the same order in the revised manuscript.

2. *“L187-190. Please provide data/figure against this observation.”*

**Response:** We mentioned that *“Cultures from liver, kidney, and vaginal samples from all of the control mice showed no growth of fungi, whereas the samples from all of the C. albicans-injected ones showed growth of C. albicans. Results of two cultures (one kidney sample from an untreated infected mouse and one vaginal sample from a mouse in arm 6) indicated contamination; they were excluded from analyses.”* The data showing the fungal colony counts in the cultures of infected mice are interpretable from the diagram presented in Figure 4; raw data is also available upon request (now mentioned in the new “data availability” section). We unfortunately did not follow a standardized manner in photographic documentation of our experiments, nevertheless, some photographs of the culture plates were taken for the record; they are now added to the supplemental contents. Our limitation in providing standardized photographic documentation of the experiments was explicitly stated in the limitations section. The contaminations we mentioned were concluded due to colonization of multiple organisms in the culture plates. This point was added to the manuscript.

3. *“L 106, was it 200 ul of suspension? Figures against the well diffusion assay need to be added.”*

**Response:** Yes, thank you for noticing our error, which is now corrected. In the supplemental contents, we uploaded a series of pictures taken for the record from the well diffusion plates; the zone of inhibition around the wells is visible in those images, but the pictures may not be considered per research standards. As mentioned, we disclosed our limitation in providing standard photographic documentation of the experiments, and also encouraged further replication following the detailed description of methods we provided, while also considering visual validation with guideline-recommended photographic documentations.

4. *“What is the reasoning behind performing the well diffusion assay when the authors performed a more sensitive broth dilution assay?”*

**Response:** We performed a qualitative well diffusion assay to have an estimate of the probable MIC measure, in order to determine the concentrations required for the more precise broth microdilution assay. Precise and objective MIC measurement was not intended to be done using zone of inhibition cut-offs in well diffusion assays, which was also the reason we did not follow the standard 24-hour incubation procedure per standard guidelines, and did not measure the MIC based on the zone diameters (as mistakenly stated in the initial draft), but visually assessed the *presence* of zones. Precise and standard MIC measurement was performed with guideline-recommended procedures using broth microdilution. These points were disclosed explicitly in the revised manuscript.

5. *“Please stick to the convention of using IFNγ.”*

**Response:** Noted. IFNg was changed to IFNγ throughout the manuscript.

6. *“What is the significance of measuring IFNg, IL10, and WBC, and ALC, What role do they play against infection?”*

**Response:** The mentioned measures were obtained as markers of extent and profile of the systemic immune response to the fungal infection. This point was clarified in the revised manuscript.

7. *“If FTY720 has antifungal activity both in vitro and in vivo then what causes fungal infections are reported among people receiving FTY720, mentioned in the introduction?”*

**Response:** We discussed and elaborated the mentioned point in the revised manuscript. Thank you once more for your time dedicated to reviewing and contributing to the coherence of our manuscript.

## Reviewer #3

0. *“The manuscript entitled “Immunomodulatory drug fingolimod (FTY720) restricts the growth of opportunistic yeast Candida albicans in vitro and in a mouse candidiasis model” is an interesting work done by the authors. However, the manuscript has several flaws starting from the way of writing to the technicality in the method section”*

**Response:** Thank you for dedicating your time to review our manuscript and highlight our errors. We hope we were able to address your comments in a sufficient manner.

1. *“The introduction lacks information about the severity of Candida albicans in present world and why authors are only targeting Candida albicans and not non-albicans Candida.”*

**Response:** Thank you for your suggestion. We elaborated more on the severity of *Candida albicans* infection in the present world, and the relevance of determining the effect of FTY720 on *Candida albicans* in our experiment.

2. *“The method section is very confusing and should be re-written for better understanding of readers.”*

**Response:** Thank you for mentioning this important point. To ensure correct understanding of the readers and reproducibility of our work, we have reworded the methods section, this time aiming to choose our words in the least confusing manner, while providing as much detail as possible.

3. *“All the methods lack relevant references”*

**Response:** Thank you for noticing. Several relevant references were added to the methods section per your request. Please do not hesitate to let us know more specifically if we are still missing any essential referencing.

4. *“Line number 106, should be rechecked for the volume, 200 ml is not correct.”*

**Response:** Indeed. Thank you for noticing. The mentioned error was corrected.

5. *“Line number 108, justification for 30-hour incubation which is not as per standard guidelines.”*

**Response:** Thank you for asking. We did not aim to measure the MIC based on the guideline-recommended zone diameter cut-offs after a 24-hour incubation of the well diffusion plates. We performed a qualitative well diffusion assay in order to estimate the probable MIC and obtain the concentration measures required for the more precise broth microdilution assay. Therefore, as only qualitative visibility of inhibition zones was of value, we incubated the plates for an additional 6 hours. As interpreted, the samples were incubated for 24 hours before precise measurement of MIC, as per EUCAST guideline for the broth microdilution assays. These points were disclosed in the revised manuscript to prevent any confusion.

6. *“Section 2.3, which labelling technique was used, which program was used and what was the basis for dose determination.”*

**Response:** The mice were labeled (ie ID-marked) using ear tags. The NumPy package for Python was used for generating a random sequence. For dose determination we decided to obtain a value frequently used in previous studies and proven to have immunomodulatory effects while being safe for the C57BL/6 mice. All of these points were disclosed in the revised manuscript.

7. *“Section 2.3, is it possible to withdraw 20 ml of blood from the mice (line 133).”*

**Response:** Certainly not. We sincerely apologize for and corrected the mentioned error.

8. *“Use abbreviations for Sabouraud dextrose agar and other reagents after mentioning at place.”*

**Response:** Thank you for your suggestion. We used abbreviations for the reagents after their first mention.

9. *“Statistical analysis should be re-written for proper understanding”*

**Response:** Along the other parts of the methods section, the statistical analysis section was reworded to ensure proper understanding of the readers.

10. *“Section 3.2, line 194, broth dilution assay should be replaced by microbroth dilution assay.”*

**Response:** Thank you for mentioning. We replaced the term “broth dilution” with “microbroth dilution”.

11. *“The results need to be discussion with reference to the relevant literature.”*

**Response:** Per your suggestion, the relevant literature was reviewed and discussed more extensively.

12. *“The manuscript lacks conclusion.”*

**Response:** A “conclusion” section was added at the end of the manuscript. Thank you sincerely for reviewing our manuscript and providing your critical appraisal of our work; your contribution is very much appreciated.
