# Supplementary material for: Immunomodulatory drug fingolimod (FTY720) restricts the growth of opportunistic yeast Candida albicans in vitro and in a mouse candidiasis model
Source: PLoS One. 2022 Dec 7;17(12):e0278488. doi: 10.1371/journal.pone.0278488 (PMC9728862; doi:10.1371/journal.pone.0278488)
Supplement: Attachment — Submitted filename: comments 26 August 2022.docx [file pone.0278488.s004.docx]

**Immunomodulatory drug fingolimod (FTY720) restricts the growth of opportunistic yeast Candida albicans in vitro and in a mouse candidiasis model**

Manuscript Id: PONE-D-22-15968

Comments after review the revised file:

1. Scientific notation of writing *Candida albicans* is not correct in abstract. Please check. It should be uniform throughout the manuscript.
2. Please incorporate the images of well diffusion assay in the manuscript.
3. Authors should keep space between values and their units throughout the manuscript and

used SI units.

4. Authors are advised to incorporate images to get better picture of their results.
5. Please incorporate toxicity related studies in the manuscript. If already published by others cite that reference in the manuscript.
