# Supplementary material for: Immunomodulatory drug fingolimod (FTY720) restricts the growth of opportunistic yeast Candida albicans in vitro and in a mouse candidiasis model
Source: PLoS One. 2022 Dec 7;17(12):e0278488. doi: 10.1371/journal.pone.0278488 (PMC9728862; doi:10.1371/journal.pone.0278488)
Supplement: Attachment — Submitted filename: Res2Rev2.docx [file pone.0278488.s005.docx]

Response to the reviewer comments

Dear Editor

Thank you for having our manuscript reviewed for the second round. We carefully considered each of the reviewer comments, revised and improved our manuscript accordingly. The changes were tracked in a marked version of the revised manuscript, which is uploaded along the clean version. Below, you can find the reviewer comments, along with our responses below each. Please let us know if any further action is required from us. Thank you in advance for your consideration; we hope to hear from you soon.

Best regards

Nahad Sedaghat on behalf of the authors

## Reviewer #1:

1. Scientific notation of writing Candida albicans is not correct in abstract. Please check. It should be uniform throughout the manuscript.

Response: Thank you for noticing. We uniformly changed all the instances in the abstract to “*Candida albicans”.*

2. Please incorporate the images of well diffusion assay in the manuscript.

Response: Thank you for your suggestion. All eFigures were moved to the main manuscript.

3. Authors should keep space between values and their units throughout the manuscript and used SI units.

Response: Thank you for mentioning. We added the required space between the values and units. About using SI units, as far as we checked, we did not use non-SI units anywhere in the manuscript. In case we overlooked an instance of using any non-SI unit, we would appreciate if you could point to the line, so we could correct that instance.

4. Authors are advised to incorporate images to get better picture of their results.

Response: Thank you for your suggestion. As mentioned, we moved all the eFigures to the main manuscript. We unfortunately have a limitation in presenting further images apart from what is already presented, as the experiments are no longer in progress. We mentioned this point in the limitations section and advised future studies to account for this limitation.

5. Please incorporate toxicity related studies in the manuscript. If already published by others cite that reference in the manuscript.

Response: Thank you for your attention. We added and discussed toxicity related studies as well in the revised manuscript. We hope the changes and improvements satisfy your concerns.

## Reviewer #2:

0. The authors have answered have all the questions raised by me.

Response: We are pleased to hear that. Thank you for dedicating your time to our manuscript.

1. However, as the authors mentioned in the article that there are some significant limitations to the reproducibility of the experiments.

Response: Thank you for mentioning your concern about the replicability of the study and reproducibility of the results. In case your concern is due to the lack of standard photographic documentation of the experiments and results, we made the following efforts to account for this limitation and make our report of merit for publication. First of all, we described and disclosed all of the experiments in full detail and in complete accordance with the EQUATOR guidelines, in order to allow their complete repetition by any scientist across the globe. Therefore, replication of the experiments and reproduction of the results would be possible for any team concerned about the lack of proper photographic documentations in the present report. Additionally, all of the experiments were performed in compliance with strict regulations and were being monitored through surveillance systems by independent scientific, safety, and ethical review boards, consisting of anonymous nationally-certified scientists. Finally, we shared and deposited our data for all researchers across the globe to review and validate. We would also welcome any further suggestions to improve our work.

2. The supplementary figures are also not as per scientific standards.

Response: Thank you for mentioning your concern. We completely agree that the presented figures are not in compliance with scientific standards, as we unfortunately, did not intend to keep scientific photographic documentations at the beginning. The reason was that in presence of strict regulations and standard guidelines, we erroneously considered photographic documentations to be optional. Now, as the experiments are no longer in process, we are unfortunately limited in terms of presenting standard figures. However, describing the experiments with full detail allows their repetition by any researcher concerned about the reproducibility of the results. In the limitations section, we explicitly stated this point, and strongly encouraged further replication of the experiments using the detailed description of the methods we provided. We further stated that due to our limitation in providing standard figures, our conclusions are subject to reproduction of the results by future studies with standard photographic documentations.

3. especially handling microbiological Petri dishes without gloves is a serious concern.

Response: Thank you for your punctiliousness. We completely acknowledge your concern. We should mention that the photograph you are referring to was taken *after completion of the experiments and documentation of their results*. *During* the experiments, the laboratory researchers followed strict regulatory standards in terms of their safety; microbiological Petri dishes were not handled without gloves during any of the experiments, and the laboratory researchers wore sterile gowns, protective face-shields and masks, as well as following standard hand-washing protocols with povidone iodine solution before entering the laboratory. This has been evidenced by absence of biological and non-biological contamination in all of the Petri dishes except for one, and by the approval of the safety procedures by the regulatory scientific review board. These points were clarified in the revised manuscript and in the legend of the mentioned figure to appreciate any concern.

4. There are still a few typos in the manuscript (eg. Line 63 homo-, IFNg instead of γ in the etable etc.).

Response: We apologize for overseeing the mentioned typos in our reviews. The Latin phrase “*in homo*” means “in man” in English; we exchanged “*in homo*” with the standard English term “in humans” to avoid any confusion. IFNg was changed to IFNγ in the supplementary table as well. Furthermore, we reviewed the revised manuscript several more times to ensure proper usage of language for the readers. We would certainly appreciate any further suggestions for improving our report.

5. Therefore, I recommend to resubmit the article with proper figures.

Response: Thank you for your recommendation. As mentioned previously, the experiments are no longer in progress, therefore, we are unfortunately limited in presenting standard photographic documentations. We truly hope our explicit and detailed disclosure of the experimental procedures according to the EQUATOR guidelines and encouragement of future replicative studies could at least, partially resolve your concerns about the reproducibility of the results. We are also, hoping that the publication of our study, if considered of merit based on novelty and relevance, leads to conduction of further studies on the subject accounting for our limitations (including provision of standard figures) and validating the results. Once again, we would like to appreciate your punctiliousness and explicitly in critically appraising our study, and thank you for your time and consideration.
