# Supplementary material for: Immunomodulatory drug fingolimod (FTY720) restricts the growth of opportunistic yeast Candida albicans in vitro and in a mouse candidiasis model
Source: PLoS One. 2022 Dec 7;17(12):e0278488. doi: 10.1371/journal.pone.0278488 (PMC9728862; doi:10.1371/journal.pone.0278488)
Supplement: Attachment — Submitted filename: Plos comments 23 Oct 2022.docx [file pone.0278488.s007.docx]

**Immunomodulatory drug fingolimod (FTY720) restricts the growth of opportunistic yeast Candida albicans in vitro and in a mouse candidiasis model**

Manuscript Id: PONE-D-22-15968

Comments after review the revised file:

1. The annotation of figures is not explicit enough, for example in the legend of figure 2 and 5, no information is provided for the images such as what type of images these are, how processed and the conditions. Please mention.
2. Quality of all disc/plates images is not good. Zone of inhibition is not visible in Figure 2 b and c. Better to crop the image, put it on black or dark background then take the image.
3. Authors are advised to thoroughly check the manuscript for grammatical errors and formatting.
